# The impact of inorganic salts on the ultrasonic degradation of contaminants: A review

**DOI:** 10.1016/j.ultsonch.2024.107076

**Published:** 2024-09-20

**Authors:** Haleigh A. Fernandez, Linda K. Weavers

**Affiliations:** Department of Civil, Environmental, and Geodetic Engineering, The Ohio State University, Columbus, OH 43210, United States

**Keywords:** Inorganic salts, Salting-out effect, Sonolytic reactors, Mass transfer, Sonochemical activity, Radical species

## Abstract

This comprehensive review explores the interplay between inorganic salts and ultrasound-assisted degradation of various contaminants. The addition of salt to aqueous matrices has been attributed to increasing contaminant degradation via the salting-out effect. However, research investigating the impact of salt on degradation has yielded inconsistent results. This review incorporated degradation information from 44 studies organizing data according to compound class and ionic strength to analyze the impact of inorganic salts on cavitation bubble dynamics, contaminant behavior, radical species generation, and contaminant degradation. Frequency and salt type were assessed for potential roles in contaminant degradation. The analysis showed that high intensity ultrasound was most beneficial to degradation in salt solutions. Unexpectedly, hydrophilic compounds showed marked enhancement with increasing ionic strength while many hydrophobic compounds did not benefit as greatly. Based on the collected data and analysis, enhanced degradation in the presence of salt appears to be primarily radical-mediated rather than due to the salting-out effect. Finally, the analysis provides guidance for designing sonolytic reactors for contaminant degradation.

## 1 Introduction

Ultrasound is an emerging technology for the degradation of contaminants due to its ease of use and lack of additional chemicals needed to achieve at least partial degradation [1]. Ultrasound degrades a diverse range of contaminants including pharmaceuticals, polycyclic aromatic hydrocarbons, dyes, phenols, and per- and polyfluoroalkyl substances (PFAS) [1], [2], [3], [4]. Degradation rates of these compounds have been shown to be significantly impacted, positively or negatively, by matrix constituents found in matrices such as wastewater, seawater, and natural water [5], [6], [7], [8], [9], [10], [11]. One such constituent that is present in all these environmental matrices is inorganic salts. The impact of salts on degradation of a target contaminant is often neglected.

Generally, while the addition of ion forming salts is not necessary, it is thought that the presence of salt will increase degradation due to the salting-out effect [12], [13], [14]. However, research investigating the degradation of contaminants in the presence of salt has been inconsistent. Seymour et al (1997) reported an increase in the degradation of phenol, chlorobenzene, and 4-ethylphenol with the addition of NaCl [12]. However, Uddin et al (2016) reported a decrease in degradation of phenol in NaCl solutions [6]. The inconsistency in results extends to other contaminant types. Research investigating the impact NaCl and CaCl_2_ have on the degradation of polycyclic aromatic hydrocarbons (PAHs) found an increase in degradation at the lowest salt concentrations studied but a decrease in degradation occurred at the highest salt concentrations [8], [15]. Yet another study showed no effect on the ultrasonic degradation of PAHs [7]. Thus, it seems that attributing the presence of salts to the salting-out effect is an oversimplification. Therefore, additional factors must be considered when elucidating the role of salts in the ultrasonic degradation of contaminants in solutions containing salt.

Due to the inconsistency in results, the aim of this review is to examine the wide body of literature surrounding the ultrasonic degradation of contaminants in the presence of inorganic salts. The goal is to provide insight into the interplay between the salting-out effect, competition kinetics, and ultrasonic conditions. Without understanding how inorganic salts impact an ultrasonic system, a large-scale system may be inappropriately designed. Therefore, by analyzing the role of inorganic salts and identifying opportunities for further research, the impact that salts have on degradation may be incorporated into designing ultrasonic systems for degradation. This review is broken down into the following four components that salt can impact in ultrasonic systems: cavitation bubble dynamics, behavior of compounds, generation of radical species, and finally an analysis of relevant research to draw conclusions on the impact of salt and opportunities for incorporating salt into design of sonolytic reactors for the degradation of contaminants.

### 1.1 The role of salt on cavitation bubble dynamics

For contaminant degradation, due to the formation of cavitation bubbles, ultrasonic frequencies of 20 kHz to, typically, 1 MHz are used. As ultrasonic waves propagate through a solution, cavitation bubbles are produced through existing nuclei (e.g., dissolved gases or suspended particles) in solution. At a certain size, cavitation bubbles rapidly expand and collapse generating a “hot-spot” where local temperatures have been estimated to reach 4000 to 10000 K and pressures up to 500 atmospheres [16], [17]. As the heat and gaseous contents transfer from the collapsed bubble, there are three reaction zones: 1) inside the bubble 2) the bubble interfacial region and 3) the solution bulk [17]. At collapse, water vapor and gaseous contents trapped in the bubble are thermally decomposed generating reactive species such as ^•^H and ^•^OH and plasma [18]. Hydrated electrons have additionally been proposed to be generated during collapse and be present for short lifetimes [19], [20], [21] In the bubble interfacial area, high temperatures also occur while generated radicals and other species diffuse from the bubble interior to the bulk solution [17]. Where the reacting compound is in relation to the cavitation bubble is dependent on the physical–chemical properties of the compound.

Inorganic salts impact cavitation bubble dynamics (i.e., formation, expansion, and collapse of cavitation bubbles) because inorganic salts increase the surface tension, increase viscosity, and decrease the vapor pressure of water [12]. For example, when the viscosity of the solution is large, cavitation is more difficult to produce [22]. Further, the Laplace pressure (ρ = 2S/R), is crucial; the Laplace pressure becomes large as bubble radius (R) decreases. Thus, there is a critical bubble radius in which bubbles smaller than it will not grow explosively due to the surface tension [23]. Therefore, the increase in surface tension resultant from inorganic salt addition hinders cavitation bubble formation, especially with small bubbles due to increased Laplace pressure. High frequency ultrasound produces smaller bubbles when compared to low frequency ultrasound [23]. Thus, the changes in surface tension due to inorganic salt introduction is more important for high frequency ultrasound. Increased vapor pressure lowers bubble collapse temperature [17]. Adding salt decreases vapor pressure [24], raising collapse temperatures during bubble collapse. Further, less water vapor will be in the bubble thereby impacting the concentration of ^•^OH produced during collapse. Studies often neglect these effects as they are difficult to measure [6], [25]. In all, these changes in water brought about by inorganic salts result in changes to sonochemical activity and corresponding rates of contaminant degradation.

### 1.2 Bubble coalescence, size, and population in salt matrices

The introduction of inorganic salt inhibits bubble coalescence and decreases cavitation bubble size. Bubble coalescence is detrimental in sonochemical systems. As bubbles coalesce and combine, the larger bubbles formed may become sonochemically inactive and dissolve out of solution [26]. Various salts, up to 3 M, have been investigated for their effect on coalescence and size [27], [28], [29]. Coalescence reduction up to 20 % has been observed at 1 M salt concentrations [28].

A strong correlation between bubble size and gas solubility has been observed in the presence of salt [27], [29]. Particularly, as gas solubility decreased, bubble size decreased. Browne et al. (2011) speculated that due to the presence of salt there is a reduction in gas content in the liquid resulting in decreased nuclei for cavitation bubble formation [27]. Indeed, gas saturation prior to experimentation and continuous gas bubbling have shown differences in bubble size with the presence of salt. Generally, bubble size becomes smaller as the frequency increases [23]. However, investigations in various salt solutions using 355 kHz [29] ultrasound reported smaller cavitation bubbles than a similar investigation using 515 kHz ultrasound [28]. The discrepancy was attributed to the difference in the gas introduction to the solution [29]. By continuously bubbling gas, nuclei will be continuously introduced resulting in smaller bubbles. Therefore, as gas is salted out by the introduction of salt, bubbles only grow to a small size compared to without salt [29].

The introduction of surface-active solutes into salt solutions further impacts bubble behavior. Salts decrease the extent of coalescence; surfactants and alcohols similarly decrease coalescence due to electrostatic repulsion and steric repulsion [26], [27], [28], [30], [31], [32], [33]. In one study, bubble coalescence decreased as the concentration of surfactant increased regardless of the charge on the surfactant [31]; however, the addition of 0.1 M NaCl to ionic surfactant solutions increased coalescence compared to with surfactants alone [32]. In contrast, no significant effect on coalescence inhibition was observed in solutions with neutral solutes with 0.1 M NaCl addition [31]. Thus, it seems that electrostatic effects are responsible for coalescence inhibition for charged surfactants while steric repulsion plays a larger role with uncharged surfactants and solutes [31]. Interestingly, the combination of ionic surfactants with salt solutions is not as helpful in inhibiting bubble coalescence compared to either alone [30].

Overall, during sonolysis, the addition of salt decreases bubble coalescence, decreases bubble size, and reduces gases and water vapor content in collapsing bubbles. Provided gas is continually introduced to maintain bubble nuclei, these factors maintain an active bubble population, reduce scattering of sound in reacting solutions and increase the collapse temperature of collapsing bubbles. Therefore, provided the effect is large enough to be observed in sonochemical degradation, enhancement in degradation is expected due to the effects of salts on cavitation bubble formation and collapse dynamics.

### 1.3 The salting-out effect

The salting-out effect occurs when the introduction of inorganic salts decreases the aqueous solubility of the contaminants [34]. Inorganic ions such as Na^+^, K^+^, Mg^2+^, Ca^2+^, Cl^-^, HCO_3_^–^, and SO_4_^2-^ decrease the solubility and increase the activity coefficient for nonpolar or weakly polar organic compounds. An empirical formula was developed by Setschenow [35] relating the aqueous solubility of a compound in pure water to that of a salt solution:

(1) $$\log\frac{C_{iw}^{sat}}{C_{iw,salt}^{sat}}=K_{i}^{s}{\left[salt\right]}_{tot}$$

where $C_{iw}^{sat}$ is the compound solubility in pure water, $C_{iw,salt}^{sat}$ is the compound solubility is a salt solution, K_i_^s^ is the Setschenow constant, or the salting-out constant, and ${\left[salt\right]}_{tot}$ is the concentration of salt. K_i_^s^, relates the effectiveness of a salt or combination of salts to a change in the solubility of a compound. This empirical relationship between the aqueous solubility in pure water and in electrolyte solutions indicates that the solubility of a compound exponentially decreases with increasing salt concentration [36].

Increased sonolytic degradation of contaminants in the presence of salts has been attributed to the salting-out effect. The driving force of a contaminant toward the interfacial region or within the bubble is expected to be impacted by the introduction of salt. Large, nonpolar molecules have higher K_i_^s^ values when compared to smaller compounds [36]. Salting-out constants show a positive correlation with octanol–water partition coefficients (logK_ow_) [37]. Additionally, salt addition has been shown to increase Henry’s Law constants [38]. Therefore, it is expected that nonpolar compounds and compounds with high Henry’s Law constants will benefit from the introduction of salt.

### 1.4 The impact of inorganic salt on measuring sonochemical activity

The introduction of salt ions leads to difficulties determining sonochemical activity. The sonolysis of water during cavitation bubble collapse results in the generation of ^•^OH and ^•^H. Hydrogen atoms react with O_2_ present to form HO_2_^•^. These radicals react with nearby species forming secondary radical species (Table 1). Self-reaction of ^•^OH and HO_2_^•^ produces H_2_O_2_:

(2) $${OH}^{∙}+{OH}^{∙}\to H_{2}O_{2}$$

(3) HO_2_^•^ + HO_2_^•^ → H_2_O_2_ + O_2_

**Table 1:** Selected radical reactions occurring in sonochemical systems in the presence of salts and their second-order reaction rate constants.

R#	Reaction	Rate constant (M^−1^ s^−1^)	Reference
1	${\;}_{}^{∙}OH{+}_{}^{∙}H\to H_{2}O$	7.0 × 10^9^	[48]
2	${\;}_{}^{∙}OH{+}_{}^{∙}OH\to H_{2}O_{2}$	$5.5\times 10^{9}$	[48]
3	${\;}_{}^{∙}H{+}_{}^{∙}H\to H_{2}$	7.8 × 10^9^	[48]
4	${\;}_{}^{∙}OH+H_{2}O_{2}\to H_{2}O+H^{+}+O_{2}^{∙-}$	2.7 × 10^7^	[48]
5	${\;}_{}^{∙}H+H_{2}O_{2}\to H_{2}O{+}_{}^{∙}OH$	9.0 × 10^7^	[48]
6	${\;}_{}^{∙}OH+H_{2}\to H_{2}O{+}_{}^{∙}H$	4.2 × 10^7^	[48]
7	${\;}_{}^{∙}OH+HOCl\to {ClO}^{∙}+H_{2}O\;\;\;\;\;\;\;\;$	$2\times 10^{9}$	[44]
8	${\;}_{}^{∙}H+HOCl\to_{}^{∙}OH+HCl$	$5\times 10^{8}$	[44]
9	${\;}_{}^{∙}H+HOCl\to {Cl}^{∙}+H_{2}O$	$5\times 10^{8}$	[44]
10	${\;}_{}^{∙}H+HOCl\to {ClO}^{∙}+H_{2}$	$5\times 10^{8}$	[44]
11	${HO}_{2}^{∙}/O_{2}^{∙-}+HOCl\to {Cl}^{∙}+\;O_{2}+H_{2}O/{OH}^{-}$	$7.5\times 10^{6}$	[44]
12	$H_{2}O_{2}+HOCl\to HCl\;{+\;O}_{2}+H_{2}O\;\;\;$	$1.1\times 10^{4}$	[44]
13	$HCl↔H^{+}+{Cl}^{-}\;\;\;$	${pK}_{a}=-6.3$	[44]
14	${Cl}^{∙}+{Cl}^{-}↔{Cl}_{2}^{∙-}$	$8.5\times 10^{9}$	[44]
15	${\;}_{}^{∙}OH+{Cl}^{-}↔{HClO}^{∙-}\;\;\;\;$	$4.3\times 10^{9}$	[44]
16	${Cl}^{∙}+HClO\to {ClO}^{∙}+H^{+}+{Cl}^{-}\;\;$	$3\times 10^{9}$	[44]
17	${Cl}^{∙}+H_{2}O\to {HClO}^{∙-}+H^{+}$	$2.5\times 10^{5}$	[44]
18	${Cl}_{2}^{∙-}+H_{2}O\to {HClO}^{∙-}+H^{+}+{Cl}^{-}$	$1\times 10^{5}$	[44]
19	${HClO}^{∙-}\to_{i}^{∙}OH+{Cl}^{-}$	$6.9\times 10^{9}\;{s^{-}}^{1}$	[44]
20	${HClO}^{∙-}+H^{+}\to {Cl}^{∙}+H_{2}O$	$2.1\times 10^{10}$	[44]
21	${HClO}^{∙-}+{Cl}^{-}\to {Cl}_{2}^{∙-}+{OH}^{-}$	$1\times 10^{5}$	[44]
22	${Cl}_{2}^{∙-}{+}_{}^{∙}OH\to HOCl+{Cl}^{-}$	$1\times 10^{9}$	[44]
23	${Cl}_{2}^{∙-}+H^{∙}\to 2{Cl}^{-}+H^{+}$	$8\times 10^{9}$	[44]
24	${Cl}_{2}^{∙-}+{HO}_{2}^{∙-}\to 2{Cl}^{-}+H^{+}$	$3\times 10^{9}$	[44]
25	${Cl}_{2}^{∙-}+{Cl}^{∙}\to {Cl}_{2}+{Cl}^{-}$	$2.1\times 10^{9}$	[44]
26	${Cl}^{∙}+{Cl}^{∙}\to {Cl}_{2}\;\;\;\;$	$8.8\times 10^{7}$	[44]
27	${Cl}_{2}^{∙-}+{Cl}_{2}^{∙-}\to {Cl}_{2}+{2Cl}^{-}\;\;\;$	$6.2\times 10^{8}$	[44]
28	${NO}_{3}^{-}{+}_{}^{∙}OH\to {OH}^{-}+{NO}_{3}^{∙}$	$1\times 10^{6}$	[49]
29	${NO}_{3}^{-}+H^{∙}\to {NO}_{2}^{∙}+{OH}^{-}$	$4.4\times 10^{6}$	[49]
30	${NO}_{3}^{-}+{Cl}^{∙}\to {NO}_{3}^{∙}+{Cl}^{-}$	$1\times 10^{8}$	[49]
31	${HCO}_{3}^{-}{+}_{}^{∙}OH\to {CO}_{3}^{∙-}+H_{2}O$	$8.5\times 10^{6}$	[49]
32	${HCO}_{3}^{-}+{Cl}^{∙}\to {CO}_{3}^{∙-}+{Cl}^{-}+H^{+}$	$2.2\times 10^{8}$	[49]
33	${HCO}_{3}^{-}+{Cl}_{2}^{∙-}\to {CO}_{3}^{∙-}+{2Cl}^{-}+H^{+}$	$8\times 10^{7}$	[49]
34	${CO}_{3}^{2-}{+}_{}^{∙}OH\to {CO}_{3}^{∙-}+{OH}^{-}$	$3.9\times 10^{8}$	[49]
35	${SO}_{4}^{∙-}+\;{HCO}_{3}^{-}\to {H{SO}_{4}^{-}+CO}_{3}^{∙-}$	$6.1\times 10^{6}$	[49]
36	${SO}_{4}^{∙-}+\;{Cl}^{-}\to {SO}_{4}^{2-}+{Cl}^{∙}$	$3.0\times 10^{8}$	[49]
37	${SO}_{4}^{2-}+\;{Cl}^{∙}\to {SO}_{4}^{∙-}+{{Cl}^{-}}^{∙}$	$2.1\times 10^{8}$	[50]
38	${HSO}_{4}^{-}+\;{OH}^{∙}\to {SO}_{4}^{∙-}+H_{2}O$	1.7 ×$10^{6}$	[50]

which has been used as an indication of sonochemical activity [33].

Brotchie et al. measured H_2_O_2_ using a 515 kHz flat plate reactor as a metric of sonochemical activity in solutions containing NaNO_3_ and NaClO_4_ in argon and helium saturated solutions. In the presence of NaNO_3_ and NaClO_4_, the sonochemical yield of H_2_O_2_ increased compared to deionized water. They attributed the increased yield to an increase in bubble temperature during collapse [28]. Contrastingly, Pflieger et al. reported a decrease in the yield of H_2_O_2_ as the concentration of NaCl increased using 362 kHz in argon and helium [33]. The authors attributed the decreased sonochemical yields to several factors including a reduction of coalescence, decreased number of active bubbles, and a corresponding decrease in sonochemical yields. Pflieger et al. [33] suggested that other chemical reactions such as with ^•^Na and ^•^Cl resulted in a decrease in H_2_O_2_ and H_2_ yields and the increase in H_2_O_2_ yield observed by Brotchie et al. [28] to NO_3_^–^ and ClO_4_^-^ participating in the reaction with ^•^OH to form H_2_O_2_, increasing the H_2_O_2_ yields. These results show salt ions may participate in radical reactions, potentially convoluting intended observations and impacting design optimization.

### 1.5 The impact of inorganic salt ions on radical species during sonolysis

Radicals from ionic species have been observed due to reactions within the cavitation bubble and around the heated outer shell. ^•^Na and ^•^Cl have been observed to form during bubble collapse from NaCl entering the cavitation bubble via droplet injection [39] and radical species have been shown to be produced in the heated shell surrounding the bubble during collapse [40]. When ^•^Na and ^•^Cl are generated, several possible reactions with other ions or radicals may occur (Table 1). ^•^Na and ^•^Cl may scavenge other reactive species including ^•^OH and ^•^H, altering sonochemical reactivity and solution properties such as the pH of the solution [33].

While the production of ^•^Na and ^•^Cl in sonicated salt solutions is known, much less is known about the production of other reactive radicals from the sonolysis of commonly used salt ions, like HCO_3_^–^, CO_3_^2–^, SO_4_^2-^, and NO_3_^–^. As indicated in Table 1, second-order reaction rate constants of these anions with ^•^OH are similar in magnitude to those with organic contaminants and ^•^OH [41]. Moreover, the concentrations of the anions is typically much higher than the concentrations of target organic contaminants undergoing degradation resulting in the salt anion outcompeting the target contaminant for ^•^OH if the contaminants are not accumulating in the cavitation bubble or on the bubble surface.

Organic compounds have diverse structures containing multiple functional groups resulting in differential reactivity with radical species. Although anions like halides are ^•^OH scavengers, the relatively nonselective ^•^OH may be converted into a more selective oxidant by reacting with the salt anion potentially aiding in treatment. Particularly, contaminants containing electron-rich functional groups may benefit from reactive halogen species while contaminants with electron-withdrawing groups may suffer [41]. For instance, the anionic radical species, CO_3_^•-^, is produced from reactions between ^•^OH and CO_3_^2–^ or HCO_3_^–^ (Table 1). CO_3_^•-^ is a weak oxidizing radical and has a wide range of second-order reaction rate constants with organic compounds (10^3^ < *k* < 10^7^ M^−1^ s^−1^) [42]. Its selectivity results in variable results and a longer lifetime compared to the non-selective ^•^OH. Therefore, selective removal of contaminants may occur [43], [44], [45], [46]. In all, trends observed with one compound may not be observed with other compounds in the presence of anion radicals.

The importance of the salts and possible radicals formed depends on the matrix. First, in surface water or drinking water, the concentration of inorganic salt ions is several orders of magnitude lower than the concentration of inorganic salt ions in ocean water or brines. Thus, the competition of salts for reactive radicals hindering or enhancing apparent sonochemical activity will vary with the matrix. Second, as the salts may affect the pH during treatment, the speciation of the radicals and their reactivity with target contaminants may be altered.

The formation of toxic by-products due to salts participating in degradation reactions needs to be assessed. Rayaroth et al. [47] found no chlorinated by products while using ultrasound while Grebel et al. [41] found chlorinated and brominated by products with UV/H_2_O_2_. Brominated by-products are typically more toxic than chlorinated by products [41]. Thus, understanding the reaction mechanisms is essential to minimize the possibility for halogenated by-products.

## 2 Methods

Studies included in this review were collected from the Web of Science using keywords such as “ultrasound”, “ultrasound inorganic salts”, and “salt enhancement.” To expand the collection, citations from collected studies were examined for possible inclusion. A subset of the studies that met criteria were included in a *meta*-analysis. Criteria for inclusion were: (1) experiments were performed without salt to act as a control, (2) experiments were performed with the addition of salt or in matrices containing salt (i.e*.*, seawater), and (3) degradation information was reported (i.e., percent degradation and/or rate constants) and (4) salts were used that dissolve into solutions forming ions. Throughout the text, for simplicity, “salt” will be used to indicate the use of salts that are dissociated into cations and anions in solution contributing to ionic strength. Studies included are listed in Table 2 along with relevant information on experimental conditions. Literature not selected for this review included salts used for radical production (e.g. S_2_O_8_^2-^).

**Table 2:** Summary of reaction conditions and observations of select literature.

**Reference**	**Compound**	**MW (g/mol)**	**logK_ow_**	**Compound concentration**	**Frequency (kHz)**	**Power/power intensity/power density**	**Salt type**	**Salt concentration**	**Author observations/attributions of results**	**Observations or effect of salt introduction**
Findik et al [52]	Acetic acid	60	−0.17	4.99 mM	40	84 W	NaCl	0.37–1.5 M	Salting-out effect Salt decreases vapor pressure and increases surface tension promoting a more violent collapse of cavitation bubbles	Maximum degradation < 8 %. NaCl enhanced degradation.
Dukkanci et al [53]	Oxalic acid	90	−0.81	2.3 mM	40	98 W	NaCl	0.15 & 0.75 M	Salting-out effect Salt decreases vapor pressure and increases surface tension promoting a more violent collapse of cavitation bubbles	Maximum degradation < 10 % NaCl decreased rate and degradation.
Gogate et al [54]	Formic acid	46	−0.54	10.8 mM 21.7 mM	590	40 W	NaCl	0.34–1.36 M NaCl	Salting-out effect Salt decreases vapor pressure and increases surface tension decreasing bubble population Radical scavenging	Maximum degradation < 13 %.NaCl enhanced degradation.
Ferkous et al [25]	Naphthol blue black	616.5	0.82 (est)	0.008 mM	1700	Applied power: Calorimetric power: 14 W	NaCl Na_2_SO_4_ Mineral water & seawater	0.1–1.0 M	Salting-out effect Bubble coalescence alterations Secondary radicals (CO_3_^–•^)	All matrices and salt types increased rate and degradation
Goel et al [64]	Trichloroethylene (TCE) Benzene Ethylbenzene Eosin B	131.3 78.1 106.1 624.1	2.61 2.31 3.15	0.38 mM 0.64 mM 0.47 mM 20 uM	20	100 W	NaCl	0.5–2.0 M	Salting-out effect Salt decreases vapor pressure and increases surface tension promoting a more violent collapse of cavitation bubbles	NaCl enhanced the rate and degradation of all compounds
Rayaroth et al [47]	Pararosaniline Ethyl violet			10 ppm 10 ppm	350	60 W	NaCl Na_2_SO_4_ Na_2_CO_3_ NaNO_3_	0.17–1.7 mM 0.07–0.7 mM 0.094–0.94 mM 0.1–1.1 mM	Salting-out effect Secondary radicals	Na_2_CO_3_ decreased rate of ethyl violet degradation NaCl, Na_2_SO_4_, and NaNO_3_ enhanced or had a neutral effect on the rate and degradation of ethyl violet and pararosaniline Na_2_CO_3_ increased rate of pararosaniline degradation
Merouani et al [11]	Rhodamine B			0.001 mM	300	60 W	Na_2_CO_3_ NaHCO_3_ Na_2_SO_4_ Mixture	0.001–0.14 M 0.005–0.12 0.0035–0.07 M 0.035 M NaHCO_3_ (fixed) + 0.7–7 mM Na_2_SO_4_	Secondary radicals	Na_2_CO_3_ and NaHCO_3_ enhanced degradation rate at low dye concentrations Na_2_SO_4_ showed little impact on degradation and rate Mixtures of NaHCO_3_ and Na_2_SO_4_ increased rate and degradation
Merouani et al [50]	Rhodamine B	479.01		0.01 mM	300	60 W	Na_2_SO_4_	0.7–35 mM	Salting-out effect Salt decreases vapor pressure and increases surface tension promoting a more violent collapse of cavitation bubbles	All Na_2_SO_4_ concentrations increased degradation rate
Hamdaoui et al [49]	Propyl paraben Naphthol blue black Naphthol blue black Malachite green Basic red 29 Acid orange 7 Rhodamine B Basic fuchsin Naphthol blue black Propyl paraben	180.2 616.5 616.5 929 368.8 350.3 479 337.8	3.04 0.82 0.82 0.62	0.028 mM 0.0081 0.016 0.0054 0.081 0.057 0.01 0.029 0.0081–0.12 mM 0.0081–0.12 mM	300 1700 585 300 300 600 300 600 585 300	30 W 14 W 80 W 30 W 30 W 30 W 30 W 30 W 80 W 40 W	Seawater		Salting-out effect Bubble coalescence	Seawater had a neutral or positive effect on rate Increasing the initial concentration of dye in seawater inhibited degradation
Rayaroth et al [14]	Coomassie brilliant blue	854		0.01 mM	350 200–1 MHz	60 W	NaCl Na_2_SO_4_ NaNO_3_ NaHCO_3_ River water River water (using different frequencies)	0.17–1.71 mM 0.07–0.70 mM 0.12–1.2 mM 1.2 mM	Secondary radicals	All salt types showed a neutral or positive effect on degradation Degradation in river water was similar for all frequencies but inhibited using 1 MHz
Guzman-Duque et al [45]	Crystal violet	408	0.51	3 mM	800	80 W	NaCl Na_2_SO_4_ NaHCO_3_	3 & 350 mM	Secondary radicals	All salt types showed minimal effect on degradation but a negative effect on rate was observed
Hamdaoui et al [5]	Acid orange 7	350.3		0.057 mM	600	120 W Calorimetric power: 31.8 W	Natural water & seawater		No explanation of data	Both matrices had a neutral effect on degradation
Wang et al [63]	Brilliant red K	808.5		0.012 mM	20	150 W	NaCl	0.5–1.5 M	Salting-out effect Salt decreases vapor pressure and increases surface tension promoting a more violent collapse of cavitation bubbles	Rate increased as salt concentration increased
Hamdaoui et al [44]	Allura red AC	496	−0.55 (est)	0.25 mM	600	Calorimetric power: 23 W Intensity: 1.83 W/cm^2^	NaCl Na_2_SO_4_ NaHCO_3_ NaNO_2_ NaNO_3_	1 & 10 mM	Secondary radicals	All salt types decreased rate with NaNO_2_ inhibiting rate the most and Na_2_SO_4_ the least
Dominguez et al [70]	Thiamethoxam Imidacloprid Acetamiprid Thiacloprid	291.72 255.66 222.6 252.7	−0.31 0.57 0.8 1.26	1 µM	578	40 W/Lc	NaCl Na_2_SO_4_ NaNO_3_ K_2_HPO_4_ NaHCO_3_ Three different river water samples	0.1 & 1 mM	Secondary radicals Salt decreases vapor pressure and increases surface tension	Na_2_SO_4_ enhanced degradation. All other salts inhibitory
Camargo-Perea et al [9]	Acetaminophen (ACE) Cefadroxil monohydrate (CDX) Cloxacillin sodium (CXL) Diclofenac sodium (DCF) Naproxen (NPX) Piroxicam (PXC) Sulfacetamide (SAM)	151 381 457.9 318.1 230 331 214	0.46 −0.4 2.48 0.7 3.18 3.06 −0.96	3.31 µM	375	Calorimetric power: 24.4 W	Mineral water	357 mg/L HCO_3_^–^ 19.6 mg/L SO_4_^2-^ 5 mg/L Cl^-^ 3.8 mg/L NO_3_^–^ 5.7 mg/L Ca^2+^ 2.5 mg/L Mg^2+^ 2.8 mg/L Na^+^ 228 mg/L K^+^	Salting-out effect Secondary radicals	High HCO_3_^-^concentration acted as an inhibitor and an accelerator. Hydrophilic compounds benefitted from mineral water matrix most
Camargo-Perea et al [9]	Sulfacetamide (SAM)			3.31 µM			NaHCO_3_ Na_2_SO_4_ NaNO_3_ NaCl	357 mg/L HCO_3_^–^ 19.6 mg/L SO_4_^2-^ 5 mg/L Cl^-^ 3.8 mg/L NO_3_^–^		Only HCO_3_^–^ showed marked effect on degradation
Camargo-Perea et al [9]	Sulfacetamide (SAM)			0.331–331 µ M			Mineral water	See mineral water composition above		Rate enhancement with all SAM concentrations with greatest enhancement at lower concentrations
Camargo-Perea et al [9]	Cloxacillin sodium (CXL)			0.331uM-331 µM			Mineral water	See mineral water composition above		Small rate enhancement at all CXL concentrations except highest
Camargo-Perea et al [9]	Cefadroxil monohydrate (CDX)			0.331uM-331 µM			Mineral water	See mineral water composition above		Rate enhancement at all CDX concentrations except highest
Serna-Galvis et al [78]	Cloxacillin sodium (CXL) Cefadroxil monohydrate (CDX)	457 381	2.48 −0.4	40uM 40uM	375	88 W/L	Seawater		Salting-out effect	Seawater enhanced the rate of CXL and inhibited the rate of CDX
Gao et al [46]	Sulfamethazine	278.33	0.89	9 µM	800	100 W	NO_3_^–^ Cl^-^, SO_4_^2-^ HCO_3_^–^ Br^-^	5 mM 5 mM 5 mM 5 mM 1 mM	Salting-out effect Secondary radicals/radical scavenging	Only HCO_3_^–^ and Br^-^ enhanced rate and degradation
Wang et al [74]	Tetracycline	444.4	−1.3	0.04 mM	20	400 W	NaNO_3_ NaHCO_3_ Na_2_SO_4_	0.11 mM 0.12 mM 0.07 mM	Changes in pH due to salt played the largest role in degradation	NaNO_3_ and NaHCO_3_ enhanced rate while Na_2_SO_4_ inhibited rate
Nejumal et al [77]	Atenolol	266	0.16	0.01 mM	350	50 W	NaCl Na_2_SO_4_ NaNO_3_ River water	0.42 mM 0.17 mM 0.29 mM	Radical scavenging Changes in bubble potential and coalescence	All salt types negatively impacted rate and degradation
Xiao et al [79]	Fluorouracil Ibuprofen Clonidine Estriol Nifedipine Lovastatin	130 206 230 288 346 404		10uM 10uM 10uM 10uM 10uM 10uM	205	45 W/L	Na_2_SO_4_ NaHCO_3_	0.002 M 0.002 M	No explanation	Both salt types showed a neutral impact on rate
Suri et al [13]	17α-estradiol 17β-estradiol 17α-dihydroequilin 17α-ethinyl Estriol Estrone Equilin	272.4 272.4 268.3 296.4 288.4 270.4 268.3	3.67 2.45 2.16	0.036 µM 0.036 µM 0.037 µM 0.033 µM 0.034 µM 0.036 µM 0.037 µM	20	Applied: 320 W Intensity: 135 kW/m^2^	NaHCO_3_ NaCl NaCl + NaHCO_3_	10 & 120 mM 0.0017 & 0.17 M 0.17 M NaCl + 120 mM HCO_3_	Salting-out effect Salt decreases vapor pressure and increases the surface tension promoting a more violent collapse of cavitation bubbles Secondary radicals	Increased rate observed with increasing NaCl except for equilin compounds Increased rate with low NaHCO_3_ concentrations (except equilin compounds) High NaHCO_3_ inhibited rates of all compounds NaCl and NaHCO_3_ mixture generally beneficial to rates
Frontisits et al [85]	17α-ethinyl estradiol	296.4	3.67	0.37 µM	20	Calorimetric power: 46 W/L	Wastewater	pH 3 pH 7.8	Salting-out effect Secondary radicals	Neutral pH increased rate and low pH decreased rate
Sasi et al [83]	Methylparaben	152.1	1.96	0.001 mM	350	22.75 W/mL	NaCl NaNO_3_ Na_2_SO_4_ Na_2_CO_3_	100 mg/L 100 mg/L 100 mg/L 100 mg/L	Salting-out effect Secondary radicals Changes in bubble potential and coalescence	Only Na_2_CO_3_ had an inhibitory effect on rate and degradation
Sanchez-Prado et al [82]	Triclosan	289.5	4.76	0.017 µM	80	135 W	NaCl Seawater	0.16 M	Salting-out effect Inorganic species acting as extra nuclei Changes in pressure and temperature resulting from salt addition during collapse	NaCl and seawater enhanced degradation and rate
Xu et al [85]	Atrazine	215.6	2.61	0.02 mM	400 20	0.03 W/mL 0.19 W/mL	NaCl	0.2–2.0 M	Salting-out effect Increase in surface tension due to salt	Varied effect based on frequency. Enhanced rate using 400 kHz with maximal rate at 1 M NaCl. 20 kHz inhibited rate at low NaCl but enhanced rate at high NaCl concentration
Rayaroth et al [84]	Chlorophene	218.6		0.05 mM	620	40.25 W/mL	Cl^-^ SO4_2_^-^ NO_3_^–^ Anion mixture	201 ppm 19 ppm 5 ppm Mixture of Cl^-^, SO4_2_^-^, NO_3_^–^	Salting-out effect Radical scavenging	No salt type or mixture of salts affected degradation
Manousaki et al [87]	Sodium dodecylbenzene sulfonate (SDBS)	326.5		0.042 mM	80	150 W	NaCl	0.042 & 0.17 M	Salting-out effect Bubble coalescence alterations	High NaCl concentration inhibited rate and degradation
Cheng et al [10]	PFOA PFOS	414.1 500.1		0.24 uM 0.19 uM	612 354 612 & 354 612	250 W/L	NaClO_4_ NaNO_3_ NaCl NaHCO_3_ Na_2_SO_4_ Groundwater NaCl NH_4_Cl CaCl_2_ MgCl_2_	1–10 mM 1–10 mM 1–10 mM 1–10 mM 1–10 mM 5 mM 5 mM 2.5 mM 2.5 mM	Salting-out effect	NaClO_4_, NaNO_3_, and NaCl increased or had a neutral effect on PFOA and PFOS rate NaHCO_3_ and Na_2_SO_4_ increased rate for PFOA and PFOS Groundwater inhibited rate and degradation with both frequencies tested NaCl, NH_4_Cl, CaCl_2_ and MgCl_2_ showed a neutral effect
Lin et al [88]	PFOA	414.1		0.12 mM	40	150 W	SO_4_^2-^	0–117 mM	Salting-out effect Secondary radicals	SO_4_^2-^ enhanced degradation and rate
Singh Kalra et al [89]	All in a mixture: PFBS PFPeS PFHxS PFHpS PFOS PFNS PFDS PFBA PFPeA PFHxA PFHpA PFOA PFNA PFDA 4:2 FtS 6:2 FtS 8:2 FtS FOSA			Variable	700	250 W	Low TDS GW High TDS GW	Variable	Salting-out effect Electrical double layer formation around cavitation bubble Bubble coalescence Reaching critical micelle concentration	Mixture of PFAS. Low TDS groundwater increased rate generally and high TDS groundwater inhibited rate.
Phan Thi et al [43]	PFOA	414		0.12 mM	40	150 W	NaHCO_3_	0.03 M	Secondary radicals	NaHCO_3_ enhanced degradation and rate
Sponza et al [15]	Acenaphthylene Fluoranthene Benzo[a]anthracene Benzo[k]fluoranthene Benzo[a]pyrene Indeno[1,2,3-cd]pyrene	152 202 228 252 252 276	3.92 5.16 5.76 6.13 6.11 6.7	0.43 µM 0.096 µM 0.0024 µM 0.0031 µM 0.00027 µM 0.0039 µM	35	51.75 W/m^2^	CaCl_2_	9–90 mM	Salting-out effect Salt decreases vapor pressure and increases surface tension promoting a more violent collapse of cavitation bubbles	Performed in mixtures in wastewater. Increased degradation for all except Benzo[a]pyrene and Indeno[1,2,3-cd]pyrene.
Sponza et al [8]	Acenaphthylene Carbazole Benzo[k]fluoranthene Benzo[a]pyrene (mixture)	152 167 252 252	3.92 3.72 6.11 6.13	0.033 µM 0.089 µM 0.00035 µM 0.00028 µM	35	51.75 W/m^2^	NaCl	0.025–0.30 M	Salting-out effect Salt decreases vapor pressure and increases surface tension	Performed in mixtures in wastewater. Optimum degradation at 0.14 M NaCl
Sponza at al [100]	Dibenzo[a,h]anthracene Anthracene Phenanthrene Pyrene Benzo[g,h,i]perylene] (mixture)	278 178 178 202 276	6.75 4.45 4.46 4.88 6.63	0.019 µM 0.020 µM 0.70 µM 0.072 µM 0.0021 µM	35	51.75 W/m^2^	NaCl	0.025–0.30 M	Salting-out effect	Performed in mixtures in wastewater. Increased degradation as salt concentration increased with optimum degradation using 0.30 M NaCl
Psillakis et al [7]	Naphthalene Acenaphthylene Phenanthrene (mixture)	128 152 178	3.3 3.92 4.46	1.17 µM 0.987 µM 0.843	80	150 W	NaCl	1.71 M	Salting-out effect Reduced vapor pressure and increased surface tension	Minimal impact on overall degradation and decreased rate for all PAHs
Psillakis et al [102]	Dimethyl phthalate Diethyl phthalate Di-n-butyl phthalate Butylbenzyl phthalate Di-(2-ethylhexyl) phthalate Di-n-octyl phthalate	194.2 222.2 278.4 312.4 390.6 390.6	1.61 2.38 4.45 4.59 7.5 8.06	1.23 µM 1.08 µM 0.86 µM 0.77 µM 0.61 µM 0.61 µ M	80	150 W	NaCl	1.71 M	Salting-out effect Reduced vapor pressure and increased surface tension	Experiments performed in mixtures. NaCl enhanced the rate for hydrophilic compounds and inhibited the rate for hydrophobic compounds
Seymour et al [12]	Chlorobenzene p-ethylphenol Phenol	112.55 122.16 94.11		2.3 mM (avg) 0.67 mM (avg) 0.74 mM (avg)	20	130–150 W	NaCl	0.17–1.38 M	Salting-out effect Salt decreases the vapor pressure and increases the surface tension resulting in a more violent collapse of bubbles	Degradation and rate increased with increasing salt concentration. Chlorobenzene had greatest degradation (∼30 %)
Sivasankar et al [103]	Phenol Chlorobenzene Nitrobenzene p-nitrophenol 2,4-dichlorophenol	94.1 112.5 123.1 139.1 163	1.46 2.84 1.85 1.91 3.06	0.53 & 1.06 mM 0.44 & 0.88 mM 0.81 mM 0.071 mM 0.61 mM	200	100 W	NaCl	0.68 M	Salting-out effect Radical scavenging	Phenol degradation benefitted the most from salt addition but degradation < 7 %. Phenol-derivatives exhibited less enhancement
Gole et al [99]	Chlorobenzene	112.55	1.46	0.88 mM & 4 mM	20	120 W applied 45 W by calorimetry	NaCl	0.02 M	Salting-out effect	Rate and degradation increased with increasing NaCl concentration at both chlorobenzene concentrations
Uddin et al [6]	Chlorophenol Phenol Catechol Resorcinol	128 94.11 110.11 110.11	2.39 1.46 0.88 0.8	0.45 mM	200	200 W applied 16 W measured by calorimetry	Na_2_SO4 NaCl	0.25 & 0.50 M 0.23 & 0.45 M	Salting-out effect Alterations in bubble population and size	Na_2_SO_4_ increased rate. NaCl had a neutral or enhancing effect for all except phenol.
Khokawala and Gogate [106]	Phenol	94.11	1.46	5.3–21.25 mM	36 25	150 W 1000 W	NaCl	8.5–26 mM	Salting-out effect	Degradation increased as NaCl concentration increased with maximum degradation < 15 % with salt
Mahamuni et al [105]	Phenol	94.11	1.46	0.95 mM & 1 mM	22	240 W Calorimetric efficiency: 5.2 %	NaCl	0.34 M & 1.36 M	Salting-out effect	Rate increased as NaCl concentration increased
Uddin et al [104]	Hydroquinone 1,4-benzoquinone	110.1 108.1	0.59 0.2	0.45 mM	200	200 W Calorimetric power: 15 W	NaCl Na_2_SO_4_	0.217 & 0.433 M 0.217 & 0.433 M	Salting-out effect	Hydroquinone: NaCl did not affect degradation but Na_2_SO_4_ enhanced degradation. 1,4-benzoquinone: both salt types increased degradation with near mineralization with Na_2_SO_4_

PFBS: perfluorobutanesulfonic acid

PFPeS: perfluoropentanesulfonic acid

PFHxS: perfluorohexanesulfonic acid

PFHpS: perfluoroheptanesulfonic acid

PFOS: perfluorooctanesulfonic acid

PFNS: perfluorononanesulfonic acid

PFDS: perfluorodecanesulfonic Acid

PFBA: perfluorobutanoic acid

PFPeA: perfluoropentanoic acid

PFHxA: perfluorohexanoic acid

PFHpA: perfluoroheptanoic acid

PFOA: perfluorooctanoic acid

PFNA: perfluorononanoic acid

PFDA: perfluorodecanoic Acid

4:2 FtS: 4:2 fluorotelomer sulfonate

6:2 FtS: 6:2 fluorotelomer sulfonate

8:2 FtS: 8:2 fluorotelomer sulfonate

FOSA: perfluorooctanesulfonamide

Degradation information reported in the selected literature was collected to determine if there were any trends in the aggregated data not easily observed in an individual study. Data points of percent degradation and/or rate constants were collected for controls and experiments with salt. An online tool (plotdigitizer.com) was used to extract data from graphs within studies to obtain percent degradation or rate constants if they were not directly reported. The collected data was transformed into a percent enhancement:

(4) $$\mathrm{Enhancement}\left(\%\right)=\frac{{\mathrm{degradation}}_{\mathrm{salt}}-{\mathrm{degradation}}_{\mathrm{control}}}{{\mathrm{degradation}}_{\mathrm{control}}}\times 100$$

where degradation_salt_ is the extent of degradation or degradation rate constant in the presence of salt and degradation_control_ is the extent of degradation or degradation rate constant in the absence of the added salt. Of the collected data, 40 % was percent degradation directly reported in the literature or retrieved from figures, 50 % were rate constants directly reported in literature, and 10 % were rate constants we calculated.

Additionally, to evaluate effects of the added salts, ionic strength, a measure of the concentration and charge of the ions in solution, was used. The reported salt type and concentration was converted into ionic strength:

(5) $$I=\frac{1}{2}\sum_{i}C_{i}Z_{i}^{2}$$

where I is the ionic strength, C_i_ is the concentration of species, Z_i_ is the charge of the species [48]. The calculated enhancement was plotted as a function of ionic strength to identify trends in the data. Finally, relevant second-order reaction rate constants between the contaminants and certain radical species (e.g. ^•^OH) were compiled and plotted to aid in data analysis (Figures S1-S4).

**Fig. 1:**
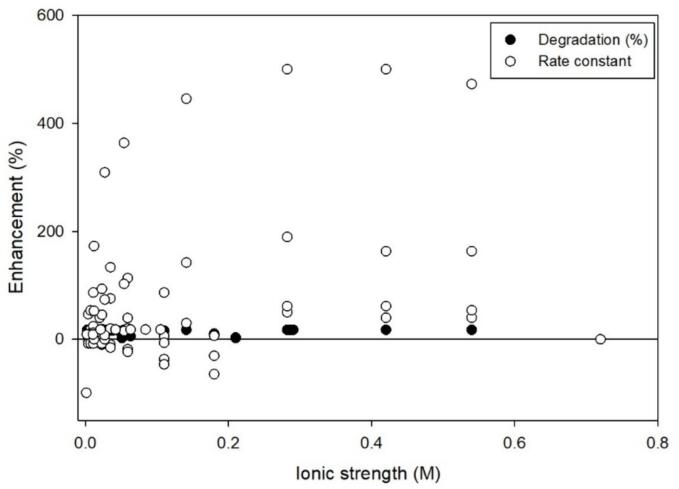
Rhodamine B degradation shown as the reported degradation (%) or rate constant. Frequency: 300 kHz. Many overall degradation (%) data points are overlayed due to similar values. Literature included: [11], [49], [50].

Because the data was analyzed in this way, there are a few caveats. First, if the percent degradation reported was low without salt (e.g*.*, 4 %) and increased in the presence of salt (e.g., 8 %), the calculated enhancement due to the small changes skews high (e.g., 100 %), distorting the apparent effect. In addition, rate constants and percent degradation were both used to calculate an enhancement. Due to varying ways to report degradation, the differing ways to assess enhancement may show different levels of enhancement for the same data. The degradation of the dye, Rhodamine B, shown in Fig. 1 is an example in which both extent of degradation and reaction rate constants were reported for the same experiments. The overall percent degradation was not markedly changed with increasing ionic strength but the corresponding reported rate constants for the same experiments increased as much as 4-fold [11], [49], [50]. Cases like these described will be noted when necessary and when available degradation rate constants are used.

The select literature and trends are presented based on the expected location of degradation: bulk liquid, interfacial region surrounding the cavitation bubble, and the gaseous interior of the bubble. The compounds included in our analysis were categorized into the following compound classes: organic acids, dyes, neonicotinoids, pharmaceuticals, endocrine disrupting chemicals, pesticides, surfactants, polycyclic aromatic hydrocarbons, semi-volatile compounds, and volatile compounds. They are generally ordered from those reacting primarily in bulk solution to those expected to react in gaseous cavitation bubbles.

## 3 Results and discussion

### 3.1 Hydrophilic compounds – Organic acids

Organic acids have low molecular weight and are hydrophilic; thus, when undergoing ultrasonic degradation in deionized water they remain in bulk solution and are degraded via reactions with OH^•^ [51], [52], [53]. The degradation of acetic, oxalic, and formic acids in salt solutions has been studied using NaCl up to 1.5 M and using a 40 kHz bath or 590 kHz flat plate system [51], [52], [53].

**Fig. 2:**
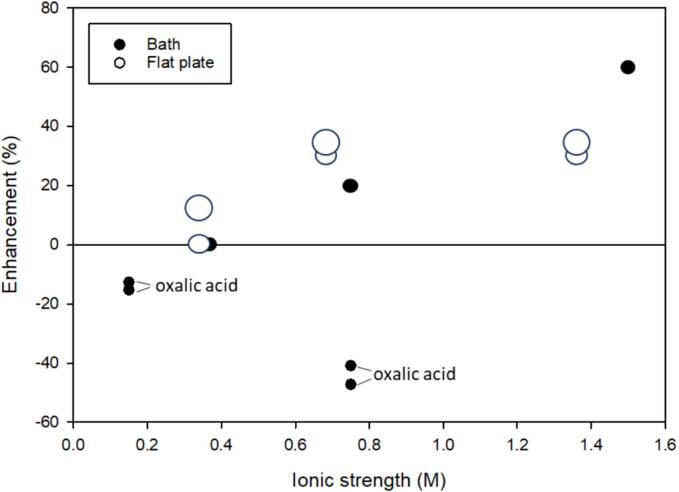
The enhancement of NaCl and ultrasonic system type on organic acids using a 40 kHz ultrasonic bath or 590 kHz flat-plate transducer. The size of the symbol indicates the relative initial starting concentration of compound. As the size of the symbol increases, the initial starting concentration increases. Only data points for oxalic acid are labeled. The remaining data points are associated with acetic acid or formic acid. References included [51], [52], [53]. Refer to Table 2 for a synopsis of each study.

As shown in Fig. 2, generally, while the extent of degradation was less than 13 %, the overall degradation of acetic and formic acids was enhanced up to 60 % with NaCl [52], [54]. As the ionic strength increased, enhancement increased for acetic and formic acids with formic acid plateauing above an ionic strength of 0.8 M. As the concentration of the organic acid increased, as indicated by the marker size in Fig. 2, enhancement also increased. In contrast, NaCl was detrimental to oxalic acid in all conditions studied [53].

Due to their negative logK_OW_ values (Table 2), radical reactions in bulk solution are the likely reaction pathway. With Cl^-^ present in solution, ^•^OH rapidly forms Cl^•^. The bimolecular rate constant of ^•^OH with Cl^-^ is 10^9^ M^−1^ s^−1^ (Table 1). Acetic acid reacts relatively quickly with Cl^•^ (k = 10^8^ M^−1^ s^−1^) [55] but rather slowly with ^•^OH (k = 10^6^ M^−1^ s^−1^) [56]. Therefore, Cl^•^ is more reactive with acetic acid than ^•^OH. In addition, as the concentration of acetic acid increased, the enhancement increased due to a better quenching rate at high concentrations. While the second-order rate constant for formic acid and Cl^•^ has not been reported, formic acid has similar characteristics to acetic acid. Therefore, we expect formic acid to react readily with Cl^•^. In contrast, oxalic acid is more strongly electron withdrawing resulting in lower reactivity with electrophilic radicals like ^•^OH and Cl^•^.

Overall, organic acids are hydrophilic, limiting their potential to salt-out and accumulate at bubble surfaces. In addition, their relatively slow reaction with OH^•^ (Figure S1) [56], [57], [58], [59], [60] and potentially faster reaction with secondary radicals results in enhancement that is compound dependent. Notably, while salt enhanced degradation for each of these compounds, overall, relatively little degradation (<15 %) was achieved, even with the addition of salt.

### 3.2 Hydrophilic compounds − Dyes

Dyes are characterized by high solubility and high molecular weight (>400 g/mol) resulting in dyes remaining in bulk solution. In the absence of salt, dyes are degraded in the bulk solution by reactions with OH^•^ [5], [14], [44], [45], [47], [49], [61]. Depending on the physical–chemical properties of the dye, degradation near the bubble interface can occur. For instance, rhodamine B can form hydrophobic dimers resulting in greater driving force to the bubble interface [62], [63].

**Fig. 3:**
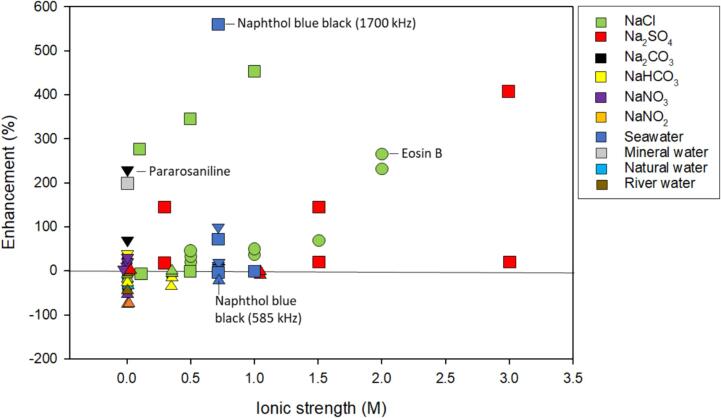
The enhancement of ionic strength, inorganic salt type, and frequency on various dyes. The symbol shape indicates ranges of frequency: ● – 20 kHz; ▾ – 200–500 kHz; ▲ – 600–1000 kHz; ■ – 1700 kHz. The color of the symbol indicates salt type as indicated by the legend. An inset of an ionic strength up to 0.012 M is available in the SI. A few dyes discussed in the text are labeled. Not all data points associated with the compounds are labeled. Data associated with Rhodamine B excluded for clarity. Included references [5], [14], [25], [44], [45], [47], [49], [61]. Refer to Table 2 for a synopsis of each study.

Several studies have investigated dye degradation in salt matrices up to an ionic strength of 3 M. Generally, like organic acids, dye degradation benefited from the introduction of salt even at high ionic strength (Fig. 3). Degradation was enhanced with NaCl, Na_2_SO_4_, and seawater (Fig. 3). The enhancement was more varied at lower ionic strengths (Figure S5).

Particularly, naphthol blue black, Eosin B, and pararosaniline benefitted from the introduction of salt. Ferkous et al. observed that NaCl, Na_2_SO_4_, seawater, and mineral water enhanced naphthol blue black degradation rate constants nearing complete mineralization of the dye. The increase was attributed to the salting-out effect and alterations in bubble coalescence [25]. Eosin B showed increased rate constants and overall degradation as the concentration of NaCl increased [64]. Pararosaniline rates benefited from the introduction of several inorganic salts such as NaCl, NaNO_3_, Na_2_SO_4_, and Na_2_CO_3_. The increases in rate constants were attributed to the salting-out effect and reactions with secondary radicals (e.g. SO_4_^-•^) [47]. On the contrary, the effect of CO_3_^2–^ introduction was not attributed to the salting out effect but rather to reactions with CO_3_^–•^ in the bulk region. Pararosaniline contains electron rich, nitrogen-containing substituents which show high reactivity toward the carbonate radical [47].

Dyes react readily with OH^•^ with bimolecular rate constants nearing the diffusion-controlled limit (Figure S1). Few bimolecular rate constants were found in the literature for other radical species derived from the salts. Therefore, it is difficult to determine if the variations in degradation were due to changes in bubble dynamics, bubble coalescence, reactions with radicals, or the salting-out effect. However, Setschenow constants increase as logK_ow_ values increase [37]. The logK_ow_ values for dyes are low [44], [65] indicating little propensity to accumulate on the bubble surface in the presence of salt ions. Therefore, dyes are unlikely to salt-out.

Hamdaoui and coworkers studied the degradation of Rhodamine B and performed a detailed analysis in Na_2_CO_3_, NaHCO_3_, and Na_2_SO_4_ solutions [11], [49], [50]. Based on Fig. 1, generally, the introduction of salts enhances the rate constant and overall degradation of Rhodamine B [11], [57], [58]. Solutions containing Na_2_CO_3_ increased the rate constants more than NaHCO_3_ or Na_2_SO_4_ individually or in mixtures [11] (Figure S6). Particularly, Na_2_CO_3_ increased the rate constants more as the starting concentration of Rhodamine B decreased. As the starting concentration of Rhodamine B increased, the impact of Na_2_CO_3_ on the rate diminished [11]. Contrary to Ferkous et al. [25], Merouani et al. [11] attributed the increase in rate to reactions with the carbonate radical instead of the salting-out effect because Na_2_SO_4_ did not impact degradation. However, bimolecular rate constants between Rhodamine B and the radicals, SO_4_^-•^ and CO_3_^–•^, were not able to be located within the literature to determine if Rhodamine B salted-out or if it was radical mediated.

Interestingly, Hamdaoui et al. [49] found that naphthol blue black enhancement was more pronounced using 1700 kHz when compared to 600 kHz but the reason for the difference between frequencies was not clear. Most ultrasonic degradation has been performed with frequencies below 1 MHz.

### 3.3 Hydrophilic compounds − Neonicotinoids

**Fig. 4:**
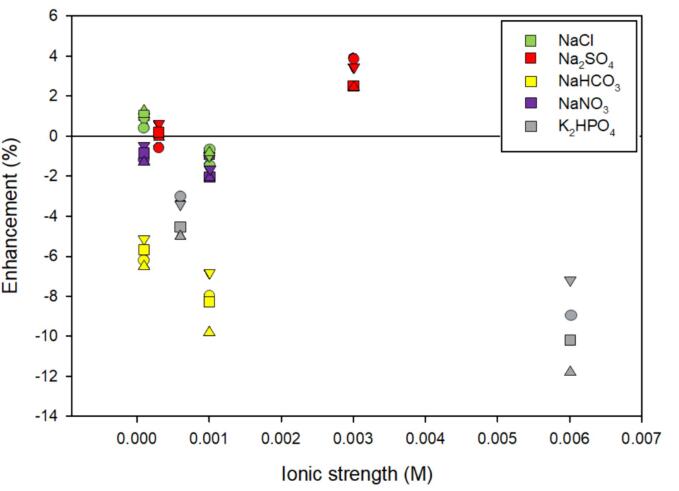
The enhancement of ionic strength, inorganic salt type, and neonicotinoid type on neonicotinoid degradation. Shape indicates specific neonicotinoid: ● – acetamiprid; ■ – imidacloprid; **▾** – thiacloprid; ▲ – thiamethoxam. The color of the symbol indicates salt type as indicated by the legend. Frequency: 578 kHz. Initial concentration: 1 uM. Included reference: [70]. Refer to Table 2 for a synopsis of the study.

Neonicotinoids are pesticides that are chemically similar to nicotine with logK_ow_ values between −0.55 and 1.26 [66], [67], [68], [69]. While high-water solubility suggest that neonicotinoids will not salt-out and react with radicals in bulk solution, Langmuir-Hinshelwood kinetics, for surface mediated reactions, were used to interpret results [70]. Generally, salt had little influence on neonicotinoid degradation. As shown in Fig. 4, decreased degradation occurred with increasing ionic strength suggesting that the addition of salt anions scavenges the available OH^•^ resulting in decreased degradation [70]. Na_2_SO_4_ is an exception. Dominiguez et al. [72] attributed the increase in degradation due to Na_2_SO_4_ to the generation of the secondary radical, SO_4_^-•^. Bimolecular rate constants for SO_4_^-•^ and acetamiprid, imidacloprid, and thiacloprid are on the order of 10^8^-10^9^ M^−1^ s^−1^ indicating SO_4_^-•^ formed effectively reacts with these compounds [71] (Figure S4). However, the bimolecular rate constant for OH^•^ and SO_4_^2-^ is 10^6^ M^−1^ s^−1^ which limits the formation of SO_4_^-•^ [58] (Table 1). The lifetime of SO_4_^-•^ is greater than that of OH^•^ (µs vs. ns) indicating that OH^•^ is more reactive and participates in reactions more than SO_4_^-•^ [72]. While acetamiprid, imidacloprid, and thiacloprid are reactive with the carbonate radical produced from OH^•^ reaction with HCO_3_^–^ and CO_3_^2–^, the rate constant is relatively small (k = 10^5^-10^6^ M^−1^ s^−1^) compared to that with OH^•^ or SO_4_^-•^ suggesting its presence will reduce degradation [73] (Figure S3). Indeed, at a similar ionic strength, Fig. 4 indicates a larger reduction in degradation for NaHCO_3_ compared to most other salts investigated.

### 3.4 Hydrophilic to moderately hydrophobic compounds − Pharmaceuticals

Pharmaceuticals contain several different functional groups resulting in difficulties determining how salt impacts degradation. Pharmaceuticals may be water soluble compounds that remain in the bulk or partition toward the cavitation bubble surface [9]. The partitioning behavior is affected by the pH of the solution altering the charge of the functional groups impacting relative hydrophilicity [9], [74].

**Fig. 5:**
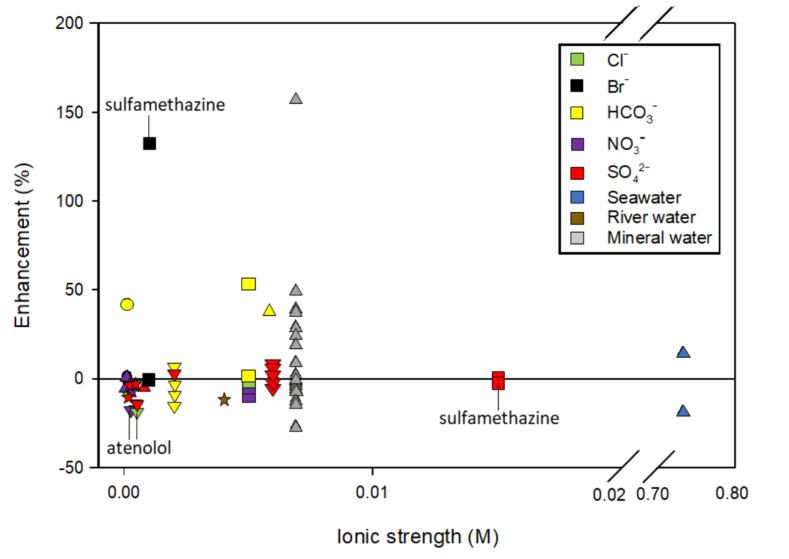
The enhancement of ionic strength, salt anion type, and frequency on the degradation of pharmaceuticals. The symbol of the marker indicates specific frequency: ● − 20 kHz; **▾** – 205 kHz; ★ – 350 kHz; ▲ – 375 kHz; ■ – 800 kHz. The color of the marker indicates salt type. Some data points associated with atenolol and sulfamethazine have been labeled. Included references: [9], [46], [74], [77], [78], [79]. Refer to Table 2 for a synopsis of each study.

When examining all the collected literature as a whole, no clear trend is observed for parameters such as molecular weight, frequency, or salt matrix. As shown in Fig. 5, salt may enhance degradation by ∼ 150 %. When the ionic strength is > 0.01 M, the enhancement of degradation trends toward zero. While trends are difficult to identify as a whole, examining individual pharmaceuticals reveals patterns.

The presence of salt from mineral water increased the degradation rates of hydrophilic compounds including sulfacetamide, acetaminophen, diclofenac, and cefadroxil [9]. Particularly, the high concentration of bicarbonate in mineral water enhanced degradation due to reactions with OH^•^ forming CO_3_^–•^. The carbonate radical has a lower recombination rate constant than OH^•^ thereby migrating in the bulk solution to act on hydrophilic substances [9]. The bimolecular rate constant of acetaminophen and CO_3_^–•^ is on the order of 10^9^ M^−1^ s^−1^ indicating high reactivity [75]. The lack of enhancement for the more hydrophobic substances was attributed to competing reactions with HCO_3_^–^ near the bubble interface [9]. Similarly, sulfamethazine degradation and rate constants were enhanced by the addition of NaHCO_3_ and NaBr which was attributed to the formation of reactive secondary radicals [46]. Indeed, the second-order rate constant of sulfamethazine and the carbonate radical is on the order of 10^8^ M^−1^ s^−1^ [76].

Other salts (e.g. NaCl, Na_2_SO_4_, NaNO_3_) were found to be less effective at enhancing the degradation of atenolol [77], sulfacetamide [9], tetracycline [74], and sulfamethazine [76]. Interestingly, the second-order rate constants for atenolol and tetracycline and Cl^•^ and are on the order of 10^8^ – 10^10^ M^−1^ s^−1^ indicating high reactivity [57]. It is unclear why these pharmaceuticals did not benefit from salt addition. Thomas and Rubino (1996) observed no significant salting-out of atenolol in NaCl solutions [37]. Therefore, the salting-out of atenolol may not be a factor in the presence of salt. This observation is consistent with the lack of enhancement by ultrasound observed in salt solutions for these compounds. Finally, seawater decreased the degradation rate of cefadroxil relative to deionized water while seawater enhanced the degradation rate for cloxacillin [78].

Camargo-Perea et al. investigated the effect of initial compound concentration of sulfacetamide, cloxacillin, and cefadroxil in mineral water dominated by NaHCO_3_ [9]. Results showed the hydrophilic compounds, sulfacetamide and cefadroxil, benefitted from almost all pharmaceutical concentrations except the highest. Cloxacillin, a hydrophobic compound, showed small positive effects at most concentrations tested except at the lowest and highest concentrations. The inhibitory effect at the highest salt concentration was attributed to the competition between cloxacillin and HCO_3_^–^ for ^•^OH as cloxacillin contains electron withdrawing group that reduces its reactivity with carbonate radicals [9]. Therefore, the influence of salts with pharmaceuticals appears to be due to radical reactions, not salting out.

### 3.5 Moderately hydrophobic to hydrophobic compounds – Endocrine disrupting chemicals

Endocrine disrupting chemicals (EDCs) included in this review are estrogen hormones, parabens, and triclosan[13]. These compounds are hydrophobic with logK_ow_ values ranging from 1.96 to 4.01 [13], [80]. Estrogen hormones and parabens have higher logK_ow_ with expected degradation in or near the bubble interface by reactionswith OH^•^ and sonolysis [13], [81]. Because these compounds are hydrophobic, they have potential to salt-out.

**Fig. 6:**
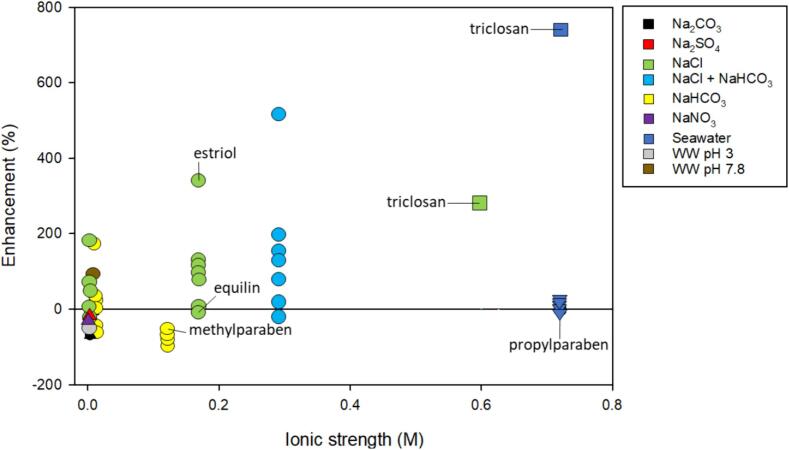
The enhancement of ionic strength, salt type, and frequency on the degradation of select endocrine disrupting chemicals including estrogen hormones, parabens, and triclosan. The shape of the symbol indicates specific frequency: ● – 20 kHz; ■ – 80 kHz; **▾** – 300 kHz; ▲ – 350 kHz. The symbol color indicates salt type as shown by the legend. Some data points associated with estriol, equilin, triclosan, and parabens have been labeled. Included references [13], [49], [81], [82], [83]. Refer to Table 2 for a synopsis of each study.

As shown in Fig. 6, degradation increases with ionic strength, especially for estrogen hormones. The presence of salt enhanced degradation by nearly 8-fold and, in some cases, salt was detrimental. Suri et al. attributed negative effects to HCO_3_^–^ scavenging of OH^•^ and positive effects to NaCl acting as a salting-out agent, neglecting the potential for Cl^•^ and CO_3_^•-^ to participate in reactions [13]. Indeed, bimolecular rate constants of 17a-ethinylestradiol and estrone with CO_3_^–•^ are relatively high (k = 10^8^ M^−1^ s^−1^) (Figure S3). A low ionic strength of NaHCO_3_ and high ionic strength of NaCl increased rate constants for estrogen hormones such as estriol but not equilins (Fig. 6). [13]. Additionally, bimolecular rate constants of the same compounds with Cl^•^ are diffusion limited (k = 10^10^ M^−1^ s^−1^) indicating high reactivity (Figure S2) [57].

Methylparaben and propylparaben generally were not altered by salt matrices. However, triclosan rate constants increased in NaCl and seawater [82]. Bimolecular rate constants for triclosan with OH^•^ and Cl radical species are all relatively high (k = 10^8^-10^10^ M^−1^ s^−1^) [57], a possible reason for the enhancement. Methylparaben was nearly completely mineralized with the introduction of salts except for NaHCO_3_. The inhibition caused by NaHCO_3_ was attributed to the scavenging of OH^•^ by HCO_3_^–^ [81].

Finally, high and low frequency ultrasound with probes and flat-plate transducers have been used for EDC degradation in salt solutions. Fig. 6 reveals that probes operating a frequency of 20 kHz and 80 kHz, as indicated by circles and squares, showed large enhancement. Flat-plate transducers at 300 kHz (at low ionic strength) and 350 kHz (at high ionic strength with propylparaben) have limited enhancement or inhibition in the presence of salts.

### 3.6 Moderately hydrophobic compounds − Pesticides

Pesticides included in this section are atrazine and chlorophene. LogK_ow_ values range from of 2.16 (atrazine) to 3.99 (chlorophene) [65] resulting in accumulation at the bubble interface with expected degradation occurring via OH^•^ reactions [84], [85]. Both compounds are hydrophobic so salting-out is expected to occur.

**Fig. 7:**
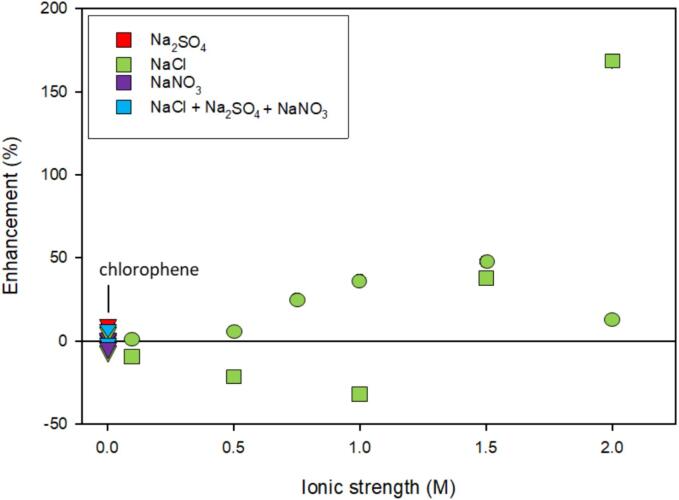
The enhancement of ionic strength, salt type, and frequency on the degradation of pesticides, chlorophene and atrazine. The shape of the symbol indicates specific frequency: ■ – 20 kHz; ● – 400 kHz; **▾** – 620 kHz. The color of the symbol indicates salt type as indicated by the legend. Some data points associated chlorophene have been labeled. Included references: [84], [85]. Refer to Table 2 for a synopsis of each study.

The presence of salt had an interesting effect on the degradation of atrazine. Using a 400 kHz flat-plat transducer, rate constants increased with increasing ionic strength until 2 M NaCl. Using an ultrasonic probe operating at 20 kHz, rate constants decreased until 1 M NaCl then rapidly increased up to 2 M NaCl (Fig. 7) [85]. The increase in rate constants was attributed to the salting-out effect [85]. Xu et al explained the decrease in rate constants to the increase in surface tension from salt addition effecting cavitation bubble nucleation and retention [85]. When rate constants decreased, the effect of surface tension was greater than that of the salting-out effect [85]. While a rate constant for atrazine and Cl^•^ was not found in the literature, atrazine has been degraded using UV/Cl suggesting atrazine is reactive with Cl^•^ [86].

Chlorophene, a halogenated phenolic compound, was investigated at the lowest ionic strength (Fig. 7). The introduction of salt, individually or in mixtures, did not have an impact on the degradation of chlorophene at low ionic strengths (<0.004 M); near complete mineralization was achieved in the presence and absence of salt in 40 min [84]. Additionally, there was no significant difference between the rate constants in the presence of salt when compared to deionized water (Fig. 7). Despite a high logK_ow_ value, salting-out did not seem to have a significant effect on degradation for chlorophene. Further, the scavenging of hydroxyl radicals by salt did not seem to pose an issue even though the salt was in excess compared to chlorophene [84].

### 3.7 Surface-active compounds

Five studies are included that investigate surfactant degradation [10], [43], [87], [88], [89]. Four of the five focus on PFAS degradation [10], [43], [88], [89] while the remaining study investigated sodium dodecylbenzene sulfonate (SDBS) degradation [87]. Of the PFAS work, most focus has been on perfluorooctanoic acid (PFOA) and perfluorooctane sulfonic acid (PFOS).

Surfactants are expected to adsorb into the bubble interface. Degradation during bubble collapse has been attributed to ^•^OH, thermolysis, plasma reactions, and hydrated electrons [90], [91], [92]. The amount of surfactant absorbed onto the bubble interface compared to the concentration in the bulk is characterized by its surface excess with degradation increasing with surface excess. Generally, the longer the alkyl chain length the greater the surface excess [93]. The addition of inorganic salts into surfactant solutions results in greater surface excess when compared to pure water [94]. Inorganic salts reduce the electrostatic repulsion of ionic headgroups at the interface and increases the activity of the hydrophobic tail resulting in an increased driving force for interfacial absorption [94]. Therefore, we expect surfactants to salt-out with the introduction of inorganic salt.

**Fig. 8:**
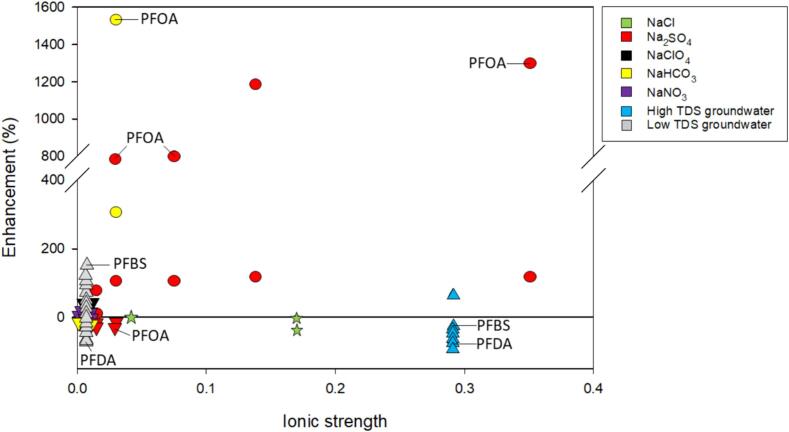
The enhancement of ionic strength, salt type, and frequency on the degradation of the surfactants, PFAS and sodium dodecylbenzene sulfonate (SDBS). Shape indicates specific frequency: ● – 40 kHz; ★ – 80 kHz; **▾** – 612 kHz; ▲– 700 kHz. The color of the marker indicates salt type as indicated by the legend. Labeled compounds: perfluorooctanoic acid (PFOA), perfluorooctane sulfonate (PFOS), perfluorobutanesulfonic acid (PFBS), perfluorodecanoic acid (PFDA). Studies included: [10], [43], [90], [91], [92]. An inset up to an ionic strength 0.016 M is available in the SI. Refer to Table 2 for a synopsis of each study.

As shown in Fig. 8, surfactant degradation was enhanced up to15-fold or inhibitory up to 1-fold. Specifically, for SDBS (indicated by ★ in Fig. 8), increasing ionic strength had a neutral effect or slight inhibitory effect on degradation and rate constants. The observed decrease in degradation at the highest NaCl concentration tested (0.17 M) was attributed to agglomeration of the surfactant from salt decreasing the critical micelle concentration [87].

Based on the five studies included in this section, there is not an obvious effect of salt on enhancement (Fig. 8). Investigations with individual PFAS, PFOA and PFOS, showed differences in rate constants and degradation when different salts were introduced and using different frequencies. Cheng et al. reported NaClO_4_, NaNO_3_, and NaCl either increased or didn’t affect the rate constant of PFOA and PFOS whereas NaHCO_3_ and Na_2_SO_4_ had an inhibitory effect [10] (Figure S7). However, similar investigations reported NaHCO_3_ and Na_2_SO_4_ increased the degradation and rate constants of PFOA [43], [88] (Fig. 8). A difference between these studies is the frequency used. High frequency ultrasound showed an inhibitory effect with Na_2_SO_4_ and NaHCO_3_ [10] but a low frequency probe showed an enhancement in PFOA degradation and rate constants [43], [88]. The increase in degradation and rate constants while using an ultrasonic probe was attributed to reactions with CO_3_^–•^ [43] and SO_4_^•-^ [88]. The bimolecular rate constant for PFOA and CO_3_^–•^ has not been identified but the bimolecular rate constant for SO_4_^•-^ and PFOA has been reported to be 10^4^ M^−1^ s^−1^ indicating low reactivity [95]. Furthermore, perfluoroalkyl substances, such as PFOA and PFOS, have been shown to not readily degrade via reactions with ^•^OH due to the strength and electron withdrawing properties of the C-F bond [96]. The most enhancement in degradation and rate constants was observed with low frequency ultrasound.

In mixtures of PFAS shown with grey in Figs. 8 and S7, short-chain PFAS degradation increased in low total dissolved solids (TDS) groundwater [89]. However, rate constants for all PFAS in the mixture decreased in high TDS groundwater (blue in Fig. 8) [89]. As TDS is comprised of salts, the surface activity increases with increasing TDS with expected increased degradation as TDS increases [97]. Further, in mixtures of PFAS, surface activity has been observed to be controlled by the most surface-active compound [94], [98]. Hence, degradation is expected to increase with increased ionic strength for all compounds, especially for the longer-chained PFAS. Inhibition in the high TDS groundwater, may be due to changes in bubble dynamics and coalescence resulting from salt addition. However, in the high TDS groundwater, Kalra et al. speculated the increase in inorganic salt ions created an electrical double layer around the cavitation bubble thereby decreasing the electrostatic repulsion between bubbles resulting in bubble collapse or coalescence [89]. Additionally, they attributed inhibition at high ionic strength to the formation of PFAS micelles similarly to SBDS.

### 3.8 Hydrophobic compounds – Polycyclic aromatic hydrocarbons

Polycyclic aromatic hydrocarbons (PAHs) are fused aromatic rings characterized by high molecular weight and high hydrophobicity (logKow > 3) [8], [15], [100]. Due to their high hydrophobicity, PAHs are expected to accumulate at the cavitation bubble interface and degrade via high temperature and reactions with OH^•^ emanating from the bubble during bubble collapse [8], [15], [100]. Salting-out constants have been reported for some PAHs ranging from 0.220 for naphthalene to 0.328 for benzo[a]pyrene [101] indicating a propensity for PAHs to salt-out.

**Fig. 9:**
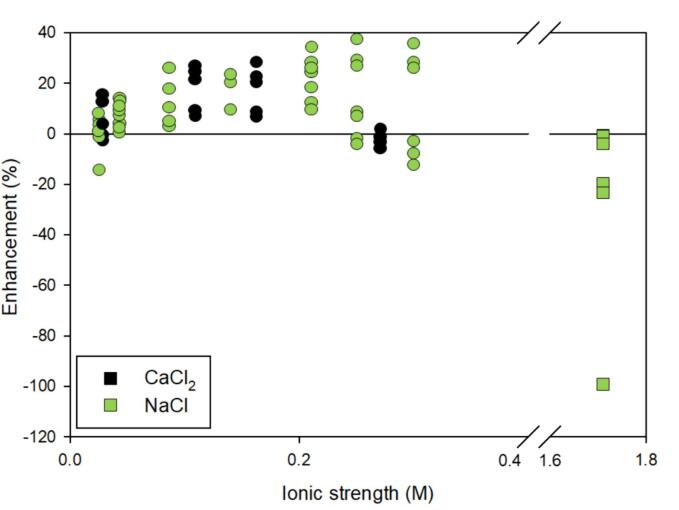
The enhancement of ionic strength, salt type, and frequency on the degradation of mixtures of polycyclic aromatic hydrocarbons (PAHs). The color of the marker indicates salt type as shown in the legend. Shape indicates specific frequency: ● – 35 kHz and ■ – 80 kHz. Included works: [7], [8], [15], [100]. Refer to Table 2 for a synopsis of each study.

A few studies have investigated the role of inorganic salts, CaCl_2_ and NaCl, on a wide range of PAHs including the volatile PAH, naphthalene, to the highly hydrophobic, benzo[a]pyrene. All four studies were conducted in mixtures with three using widely varying concentrations (nM to µM) in wastewater [8], [15], [100]. Generally, as shown in Fig. 9, with increasing ionic strength, PAH degradation was enhanced up to 40 % [7], [8], [15], [100]. At a high ionic strength of 1.71 M, Psillakis et al. observed inhibited rate constants [7]. The introduction of salt seems to drive the PAHs toward the bubble for enhanced degradation but there is a divergence in the data 0.2 M. It is unclear why the data diverges. While the ultrasonic frequency remains the same (35 kHz), the divergence in the data is difficult to interpret due to mixtures of PAHs being used, varying concentrations, and complex matrices (i.e. wastewater).

In wastewater, Sponza and coworkers found a similar optimum ionic strength for PAH degradation (i.e. 0.14–0.16 M) with both NaCl and CaCl_2_ in two studies [8], [15] while all NaCl concentrations enhanced degradation in a similar investigation [100]. At the optimum concentrations for all studies, PAH degradation was near mineralization [8], [15], [100]. The increase in degradation, especially for the less hydrophobic PAHs, was attributed to the salting-out effect. Salting-out increases with logKow; therefore, the more hydrophobic PAHs are expected to benefit the most from salt addition but this trend was not observed. Concentration differences among the PAHs in the mixtures spanning several orders of magnitude between the most hydrophobic PAHs (nM) and the least hydrophobic PAHs (uM) may play a role in results.

### 3.9 Moderate Henry’s Law constants − Semi-volatile organic compounds

Semi-volatile organic compounds (SVOCs) encompass a wide range of compounds that have moderate Henry’s Law constants. Compounds included in this review categorized as SVOCs include phenol and derivatives (e.g. chlorophenol, resorcinol, hydroquinone, etc.) and phthalate esters. LogK_ow_ values for these SVOCs range from greater than 1 to up to 8 [6], [102]. Based on the properties of the SVOCs, degradation may range from primarily in the bulk solution to in the bubble [6], [102], [103]. Because most phenolic compounds have low a logK_ow_, salting-out is not expected. However, for phthalate esters, the salting-out effect is expected to occur due to logK_ow_ values reaching up to 8 [65]. Additionally, the addition to inorganic salt has been shown to increase Henry’s Law Constants [38]. Therefore, the driving force for a compound to partition into the bubble may be increased resulting in increased degradation.

**Fig. 10:**
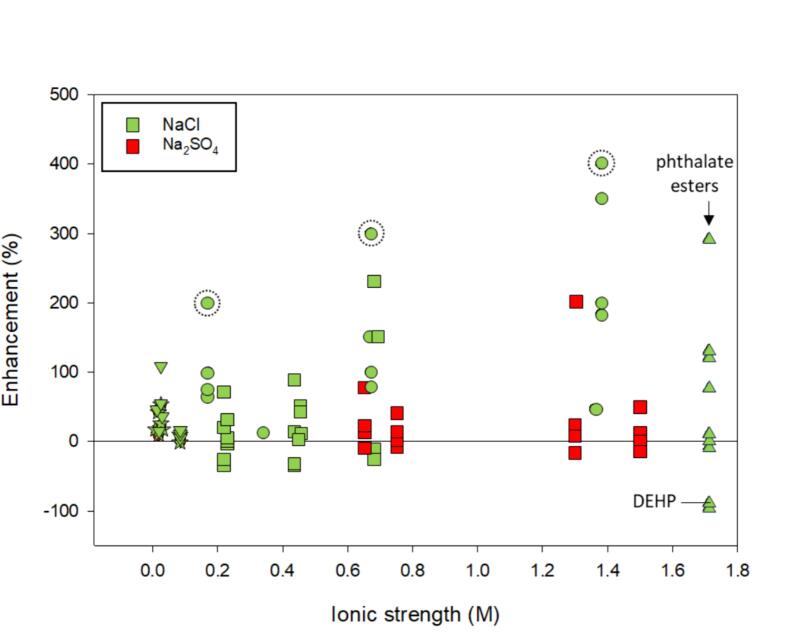
The enhancement of ionic strength, salt type, and frequency on semi-volatile compounds. The marker shape indicates specific frequency: ● – 20 kHz; ★ – 25 kHz; **▾** – 36 kHz; ▲ – 80 kHz; ■ – 200 kHz. The marker color indicates the salt type as indicated by the legend. The dashed circles indicate instances where the enhancement is distorted for 4-ethylphenol due to low initial degradation as discussed in the text. Literature included: [6], [12], [102], [103], [104], [105], [106]. Refer to Table 2 for a synopsis of each study.

Based on Fig. 10, generally, there is an increase in degradation rate for SVOCs. For most compounds, the presence of salt enhanced degradation up to nearly 4-fold while, in some cases, the introduction of salt was detrimental by up to 1-fold. Enhancement was observed with all types of ultrasonic systems (bath, probe, and flat-plate transducer) but enhancement was greatest with a probe system at 20 kHz. Uddin et al. [6] evaluated both NaCl and Na_2_SO_4_ with several individual SVOCs and was not able to attribute effects to salting-out when considering logK_ow_ and dynamic surface tension after salt addition.

**Fig. 11:**
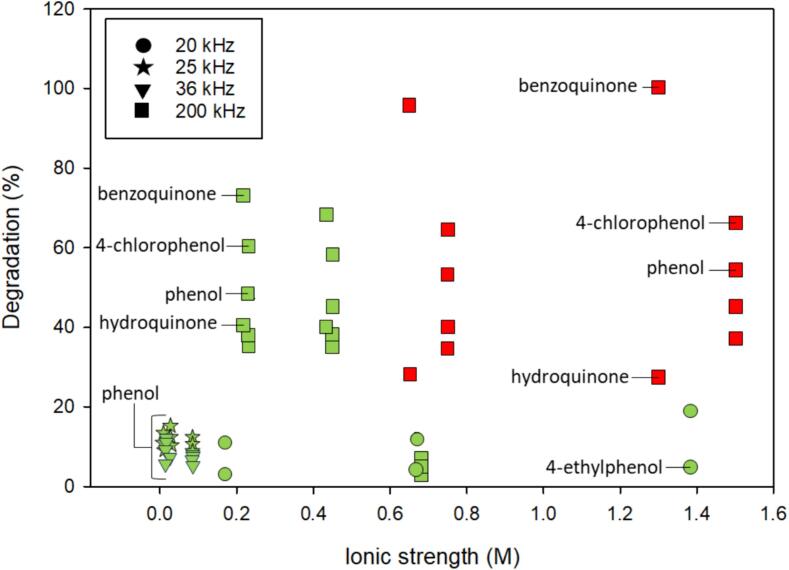
The overall degradation (%) of phenol and similar compounds in solutions containing inorganic salts. Marker shape indicates frequency as shown in the legend. Color indicates inorganic salt type: green – NaCl; red – Na_2_SO_4_. Rate constants not included. Excluding phthalate ester data. Included references: [6], [12], [104], [106]. (For interpretation of the references to color in this figure legend, the reader is referred to the web version of this article.)

While enhancement was observed with the addition of inorganic salts for phenol and similar compounds (benzoquinone, 4-chlorophenol, hydroquinone, catechol, resorcinol, 4-ethylphenol, 4-nitrophenol, 2,4-dichlorophenol), the way we analyzed the data may distort the relative enhancement. For instance, 4-ethylphenol showed the greatest enhancement (Fig. 10, three data points indicated with a dashed-circle) with degradation of 1 % in deionized water and 5 % with inorganic salt addition [12] resulting in an enhancement of 400 % but little overall degradation. To assess the role of inorganic salt addition has on the degradation of phenol and similar compounds, degradation (%) in inorganic salt solutions in addition to enhancement was evaluated (Fig. 11).

An assessment of best conditions between Fig. 10, Fig. 11 are inconsistent. Based on Fig. 11, the greatest degradation of phenol and derivatives was observed at 200 kHz over a wide range of ionic strengths. Interestingly, the compounds that showed the greatest degradation using 200 kHz (benzoquinone, phenol, hydroquinone, catechol, resorcinol) contained electron donating groups and were investigated at the lowest concentrations of the SVOCs (0.45 mM) [6], [104]. In Fig. 10, these conditions resulted in less enhancement with salt. Compared to higher frequency, degradation when using a probe or ultrasonic bath (all frequencies except 200 kHz) was < 20 %. Within these systems, concentration differences contribute to discrepancies. Uddin et al. [6] reported 50 % phenol degradation with the addition of NaCl and Na_2_SO_4_ individually whereas Sivanskivar et al. [103] found degradation to be about < 6 % with the addition of NaCl. Sivanskivar et al. [103] used at least 11-fold greater concentrations of phenol than Uddin et al [6].

Phthalate esters were degraded to near mineralization at the highest ionic strength (1.71 M) and showed increased rate constants compared to no salt, except for the most hydrophobic phthalate esters, di-(2-ethylhexyl) phthalate (DEHP) and di-n-octyl phthalate [102]. Due to high logK_ow_ values, it was expected that the most hydrophobic compounds would salt-out and increase rate constants. Although salting-out was expected, the most hydrophobic phthalate esters have high molecular weights and may be limited by diffusion.

Overall, studies investigating these phenolic-type compounds did not consider the potential for the salts to participate in reactions. When examining the salt enhancement at 200 kHz [6], [104], hydroquinone and resorcinol showed the greatest enhancement using NaCl. Other compounds including catechol, 4-chlorophenol, phenol, and benzoquinone did not benefit from salt addition. At higher ionic strengths, in solutions containing Na_2_SO_4_, inorganic salt ions either had a neutral effect or increased degradation up to 30 % (Figs. 11 and S8). While these compounds contain electron withdrawing functional groups that could inhibit degradation with OH^•^, Cl^•^, and SO_4_^-•^, Figs. S1, S2, and S4 indicate bimolecular rate constants near diffusion-controlled limits suggesting their involvement in degradation.

### 3.10 High Henry’s Law constants – Volatile compounds

Volatile organic compounds (VOCs) are characterized by low molecular weight and high Henry’s Law constants [64], [107]. They are expected to partition into the bubble interior and degrade during bubble collapse [108]. Compounds categorized as volatile in this review include benzene, chlorobenzene, ethylbenzene, nitrobenzene, and trichloroethylene (TCE). Except benzene, these VOCs contain electron withdrawing functional groups which reduce reactivity with electrophilic radicals. These VOCs exhibit logK_ow_ values of < 3.15 resulting in the propensity to salt-out [65], [103]. Ethylbenzene exhibits the greatest logK_ow_ (3.15) as well as the greatest salting out constant (0.238) [109]. Dissolved salts increase Henry’s Law constants [38].

**Fig. 12:**
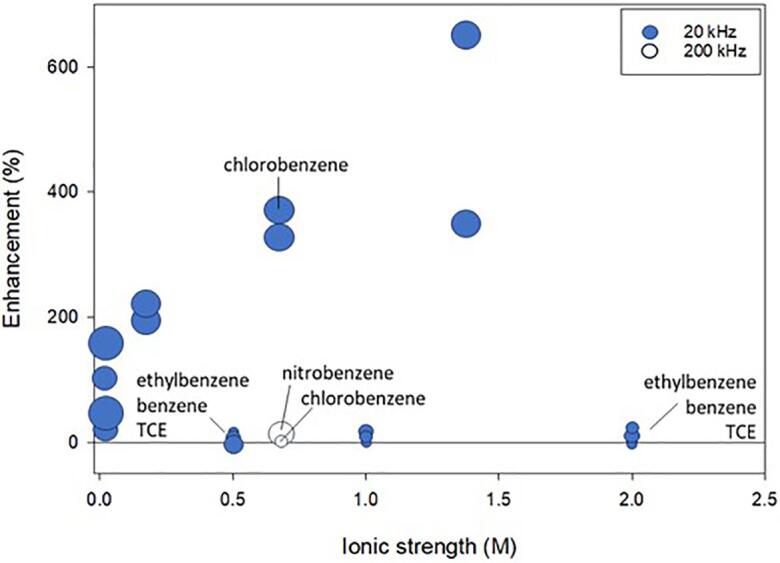
The enhancement of NaCl and frequency on the degradation of compounds with high Henry’s law constants. The color of the marker indicates specific frequency as indicated by the legend. The size of the symbol indicates the initial starting concentration of compound. As the symbol size increases, the starting initial concentration of compound also increases. A few datapoints associated with compounds discussed in the text are labeled. TCE is trichloroethylene. Included references: [12], [64], [103], [107]. Refer to Table 2 for a synopsis of each study.

As shown in Fig. 12, except for chlorobenzene, VOCs did not benefit but were not hindered by the addition of NaCl [12], [64], [103], [107]. Chlorobenzene degradation and rate constants were enhanced up to 6-fold with increasing ionic strength. Using an ultrasonic probe operating at 20 kHz, Seymour et al. reported chlorobenzene degradation up to only 30 %, the greatest enhancement of the studies investigating chlorobenzene [12].

Degradation up to 90 % was observed for chlorobenzene in the presence of NaCl using an ultrasonic bath operating at 20 kHz [107]. Contrastingly, Seymour et al. reported chlorobenzene degradation up to only 30 % using an ultrasonic probe operating at 20 kHz [12]. Similarly, Gole and Gogate observed degradation up to 90 % and a doubling in the degradation rate constant for chlorobenzene in the presence of NaCl using an ultrasonic bath operating at 20 kHz [107]. However, we note that the extent of degradation was low in Seymour and Gupta [12]. The experimental set up in Gole and Gogate suggests the ultrasonic bath and solution were open to the atmosphere, allowing volatilization [107]. Based on Fig. 12, chlorobenzene degradation in NaCl benefits from higher initial compound concentrations. However, the data may show greater enhancement than expected due to low initial degradation (i.e. 4 %) with a maximum degradation in the presence of salt being about 30 % [12].

Furthermore, chlorobenzene degradation enhancement was greater using a 20 kHz probe when compared the degradation using a flat-plate transducer operating at 200 kHz. Using 200 kHz, 70 % degradation was reported for chlorobenzene in NaCl with only a 7 % enhancement when compared to deionized water. The small enhancement using 200 kHz was attributed to chlorobenzene being at saturation at the bubble-bulk interfacial region [103]. Similarly, Goel et al. did not observe a large effect in degradation in benzene, ethylbenzene or TCE degradation [64]. Ethylbenzene has a higher logK_ow_ than chlorobenzene and trichloroethylene has a similar logK_ow_ to chlorobenzene, suggesting a mechanism in addition to or other than salting-out.

While these compounds are volatilizing into the cavitation bubbles and little effect of salts has been observed, salts may be participating in reactions. Benzene and substituted benzenes react with OH^•^ near diffusion-controlled limits (Figure S1). Benzene and chlorobenzene react with Cl^•^ similarly to other compounds where Cl^•^ is near the diffusion-controlled limit (Figure S2). Therefore, these volatile compounds will degrade by OH^•^ and Cl^•^.

## 4 Conclusion and recommendations

### 4.1 Conclusions and implications of salts in degradation

The salting-out effect has been a major source of motivation for the addition of salts to ultrasonic systems. Particularly, Seymour et al [12] published one of the first studies that investigated the ultrasonic degradation of contaminants in salt matrices with the goal of enhancing degradation and improving sonochemical efficiency. Since then, much work has been done seeking to exploit the salting-out conditions brought about by salts. Of the 44 studies included in Section 3 and Table 2 of this review, 34 indicated the salting-out effect to be a major source of motivation or explanation of results; 17 mentioned the role of radicals generated from salt addition. The results of this review show that the salting-out effect is an oversimplification of salt addition. The ultrasonic degradation of contaminants in the presence of inorganic salts does not guarantee increased degradation.

In the absence of a target contaminant, the introduction of inorganic salt ions impact cavitation bubbles in several aspects. First, the surface tension, viscosity, and vapor pressure are altered by inorganic salts, altering the formation and collapse of cavitation bubbles. Second, inorganic salts have been shown to decrease cavitation bubble coalescence and bubble size. Finally, the presence of inorganic salts complicates the measurement of sonochemical activity by participating in reactions including OH^•^ scavenging which are crucial radicals for H_2_O_2_ measurement. Inhibited coalescence prevents the bubble population from decreasing and increasing available cavitation bubbles for degradation. Contrastingly, an increase in surface tension makes cavitation bubble formation more difficult, reducing active cavitation bubbles. These changes in cavitation bubble dynamics and sonochemical activity are difficult to measure resulting in challenges in determining optimal conditions, even in the absence of target contaminants. Further, these changes have not been directly linked to observable effects on contaminant degradation.

Contaminant degradation does not always increase with salt addition as expected with the salting-out effect. For the hydrophilic compounds such as those in the categories organic acids, neonicotinoids, and pharmaceuticals, enhancement seems to be radical based rather than due to salting-out. For instance, hydrophilic pharmaceuticals benefitted from mineral water dominated by NaHCO_3_ due to reactivity with CO_3_^–•^ but the hydrophobic compounds did not benefit as greatly. If the salting-out effect was the main mechanism of degradation, then hydrophobic compounds would have benefitted the most from salt addition due to greater propensity to salt-out. Radical based degradation is further exemplified with neonicotinoids. Matrices with Na_2_SO_4_ resulted in the greatest enhancement while the addition of NaHCO_3_ inhibited neonicotinoid degradation most. Examining second-order rate constants showed high reactivity of neonicotinoids with SO_4_^-•^ and low reactivity with CO_3_^–•^. For more hydrophobic compounds, where the salting-out effect is expected to be dominate, results do not always show enhancement. In low TDS groundwater, PFAS mixtures showed shorter-chained PFAS enhancement more so than long-chain PFAS which was unexpected. Similarly, the less hydrophobic PAHs and phthalate esters benefitted from salt addition compared to the more hydrophobic compounds. Although speculative, the lack of enhancement may be diffusion related.

Further evidence for radical based degradation in the presence of salt is revealed when considering the type of ultrasonic system used (i.e. probe, bath, flat-plate). The use of ultrasonic probes seems to have the most beneficial effect on compound degradation in salt matrices (See 3.5, 3.7, 3.9). Typically, ultrasonic probes operate at high power intensity with power intensities ranging from 10 to 60 Wcm^−2^ while flat-plate transducer units and ultrasonic baths have intensities on the order of 10 to 20 times less than probes [2], [15]. The high power intensity conditions created in a probe reactor produce a dense conical-shaped bubble cloud near the probe tip resulting in scattering of the sound by the bubble and little sound penetration into the solution [110]. Moreover, the dense cloud contains interstitial fluid that enters the bubble during collapse [2]. In the presence of salt, it is plausible that more salt enters the bubble during collapse in probe systems resulting in different or a greater concentration of radicals generated. Whereas high frequency ultrasound generated with flat-plate systems and low frequency bath systems are at a low power intensity creating greater spatial distribution within the reaction solution resulting in reactions occurring throughout the entire solution without a dense bubble cloud to promote injection of interstitial fluid into the hot collapsing bubble [2]. Exceptions exist, especially with very high frequency. This system needs further exploration to understand the large enhancement observed.

Based on the analysis presented here, it appears that degradation in the presence of salt is radical mediated rather than due to the salting-out effect. If salting-out was the main mechanism by which degradation increased, then enhancement would be expected with the use of both probes and flat-plate transducers, and perhaps more prominent with flat-plate transducers. Further, the effects would not be altered by salt type. The participation of secondary radicals formed from anions reacting in cavitation bubbles or with primary radicals formed in the cavitation bubbles (e.g., OH^•^) has been largely neglected. Reactions of these radicals are both radical and compound specific and bimolecular rate constants of reactions need to be incorporated into degradation studies. Electron donating substituents aided in reactions with generated secondary radicals (e.g., Cl^•^). More studies evaluating multiple salts as a function of ionic strength on degradation are needed to elucidate the role of salts and secondary radicals in degradation.

Because our results show the degradation of contaminants using ultrasound is radical mediated with salts, measuring potential by-products is essential. Halogen radicals react with many contaminants, some at near-diffusion limits, resulting in possible toxic halogenated by-products. Other degradation technologies have shown toxic by-product formation for compound classes such as PAHs or semi-volatile organic compounds [111], [112], [113]. Few ultrasonic investigations have attempted to measure possible toxic by-product formation after experimentation in salt matrices. This should be a future priority.

### 4.2 Design recommendations

When designing large scale ultrasonic systems in the presence of salts, this review underlines the importance of considering several parameters. First, because enhancement was often observed with ultrasonic probes, degradation with high power intensity ultrasonic units may benefit more with salt addition compared to low power intensity ultrasound. This effect is especially true when considering hydrophobic compounds and surface-active compounds. Because degradation appears to be radical mediated, bimolecular rate constants between secondary radicals formed from salt and target contaminants must be incorporated into design. Depending on the physical–chemical properties of the compound, secondary radicals (e.g., CO_3_^–•^) may enhance or inhibit degradation which further emphasizes the need to consult bimolecular rate constants in solutions containing salt. Second, although toxic by-products have not been detected in investigations included in this review, other degradation technologies have detected the presence of halogenated by-products. Assessing toxic by-product formation should be a priority when designing large scale ultrasonic systems. Finally, the presence of inorganic salts complicates the measurement of sonochemical activity.

Overall, this review revealed that ultrasonic degradation of contaminants in salt solutions is more complex than simply the salting-out effect. While reported enhancements have been substantial, they may have low degradation and the degradation is much more complicated than salting-out. Therefore, to design ultrasonic systems optimally with limited costs, the reactor used, the composition of the matrix, the structure of the target compounds, and possible by-product formation must be evaluated due the generation and fate of radical species.

### 4.3 Recommendations for future work

To maximize the design of ultrasonic systems, understanding the role and fate of salts is crucial because all environmental aqueous matrices contain salt ions. It will aid in determining the appropriate solvent choice. Therefore, the role of salts on degradation via ultrasound presents a research gap. The work presented in this review is a first step in filling that gap and serve as a base to understand what research still needs to be done. First, our proposed mechanism that degradation in the presence of salt is predominately radical-mediated rather than by the salting-out effect needs to be verified. To achieve this, more research using the high-intensity ultrasonic probes in salt matrices is needed because the interstitial fluid appears important for radical generation. Second, studies on salt types other than NaCl are needed, ideally, all at same ionic strength to verify the role of secondary radicals beyond Cl^•^. Second-order rate constants for potential radical species must be incorporated as well as compound structure if kinetic data is not available. Third, experimentation with compounds individually and in mixtures is crucial to interpret how salt impacts compounds individually which can be used to explain data of mixtures. Finally, potential by-product formation needs to be a priority. Halogen radicals, especially with high intensity probes, are being generated with the potential of adding to target compounds. These halogenated by-products are toxic and need to be monitored.

In all, salt addition is much more complicated than the salting-out effect ranging from complications to measuring sonochemical yield to changes in degradation based on frequency or compound type. This review has revealed several research gaps that must be filled to fully maximize sonochemical design. Without further elucidating the role of salts, the design may not be cost effective and have unintended consequences (i.e. by-products).

### CRediT authorship contribution statement

**Haleigh A. Fernandez:** Writing – review & editing, Writing – original draft, Methodology, Data curation. **Linda K. Weavers:** Writing – review & editing, Supervision, Resources, Project administration, Methodology, Investigation, Conceptualization.

## Declaration of competing interest

The authors declare the following financial interests/personal relationships which may be considered as potential competing interests: Linda Weavers reports financial support was provided by Ohio Water Development Authority. Editorial Board for Ultrasonics Sonochemistry, LW If there are other authors, they declare that they have no known competing financial interests or personal relationships that could have appeared to influence the work reported in this paper.

## Data Availability

Data will be made available on request.
