# Supplementary material for: The impact of inorganic salts on the ultrasonic degradation of contaminants: A review
Source: Ultrason Sonochem. 2024 Sep 20;111:107076. doi: 10.1016/j.ultsonch.2024.107076 (PMC11639443; doi:10.1016/j.ultsonch.2024.107076)
Supplement: Supplementary Data 1 [file mmc1.docx]

Supplementary information

The effects of salt on the ultrasonic degradation of contaminants: a review

Haleigh A. Fernandez ^†^, Linda K. Weavers ^†^*

^†^Department of Civil, Environmental, and Geodetic Engineering, The Ohio State University, Columbus, Ohio 43210, United States

*To whom correspondence should be addressed. Phone: (614) 292-4061

email address: [weavers.1@osu.edu](mailto:weavers.1@osu.edu).


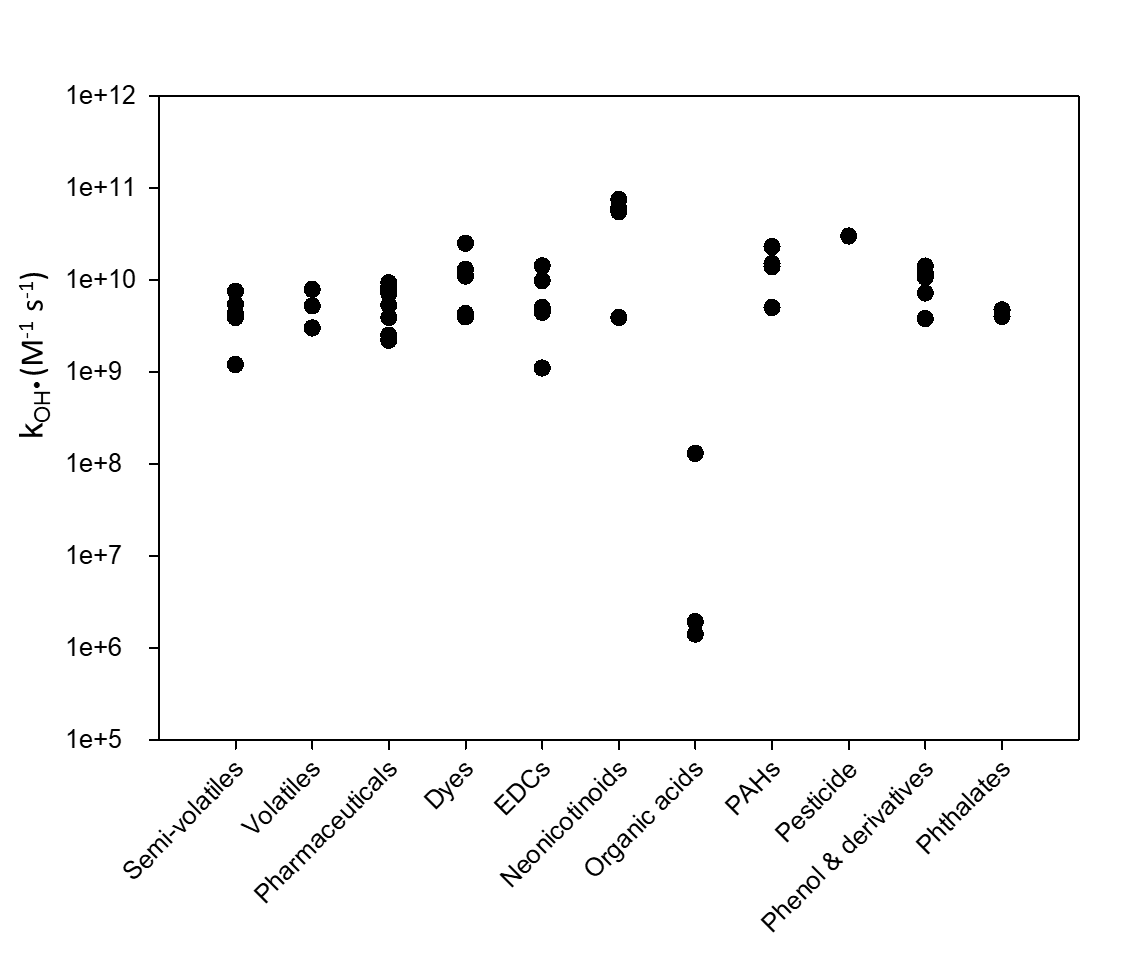


**Figure S1.** Second-order rate constants for compounds included in this work and ^•^OH. The rate constants are categorized into similar categproes as presented in the main body of the text. Dyes: [1–3]; endocrine disrupting chemicals (EDCs): [4–8]; neonicotinoids: [9,10]; organic acids: [2,11,12]; polycyclic aromatic hydrocarbons (PAHs): [2,13,14]; Pesticide: [15]; Phthalates: [7,16]; pharmaceuticals: [5,7,17,18]; Semi-volatiles: [2,19–21]; Volatiles: [2,5,22,23].


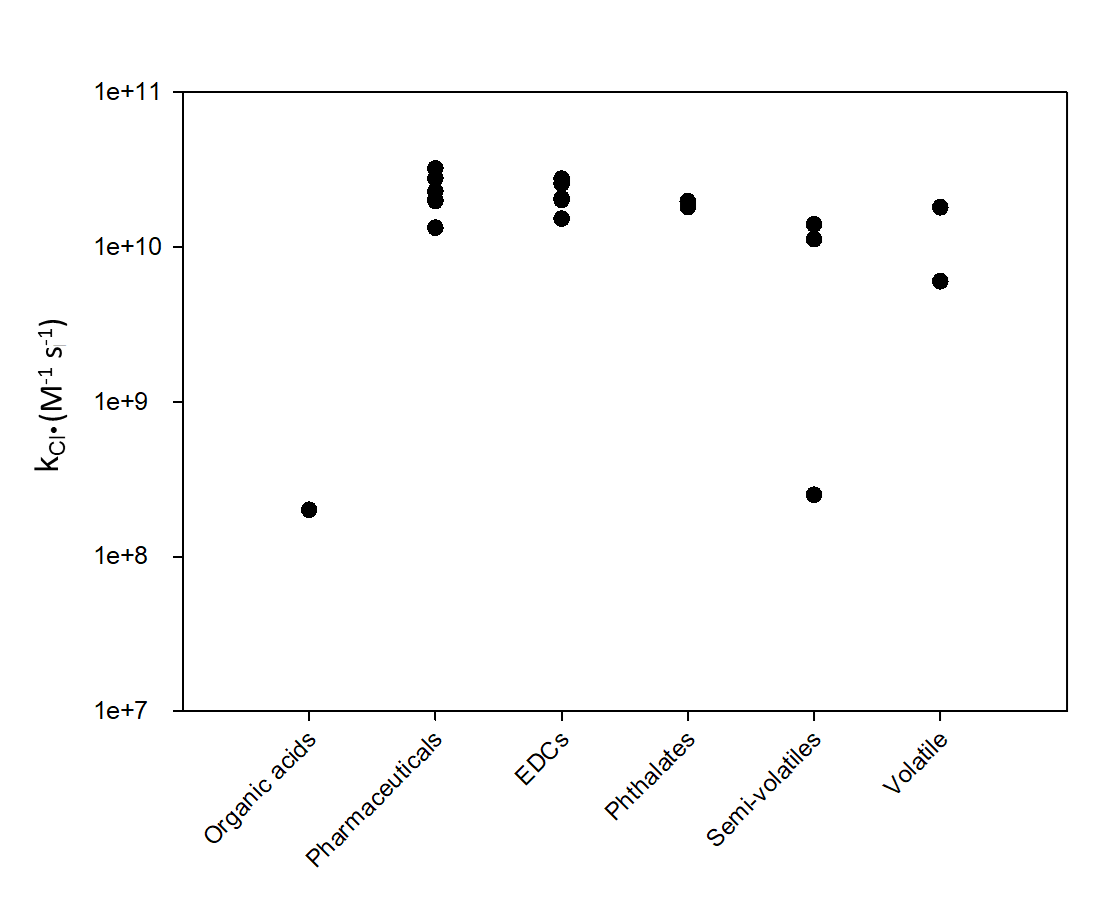


**Figure S2.** Second-order rate constants for compounds included in this work and Cl^•^. The bimolecular rate constants for the compounds are categorized into the same categories as presented in the main body of the text. Organic acid: [24]; pharmaceuticals: [7,18]; endocrine disrupting chemicals (EDCs): [7]; phthalates: [7]; semi-volatiles: [7,25,26]; volatile: [27,28].


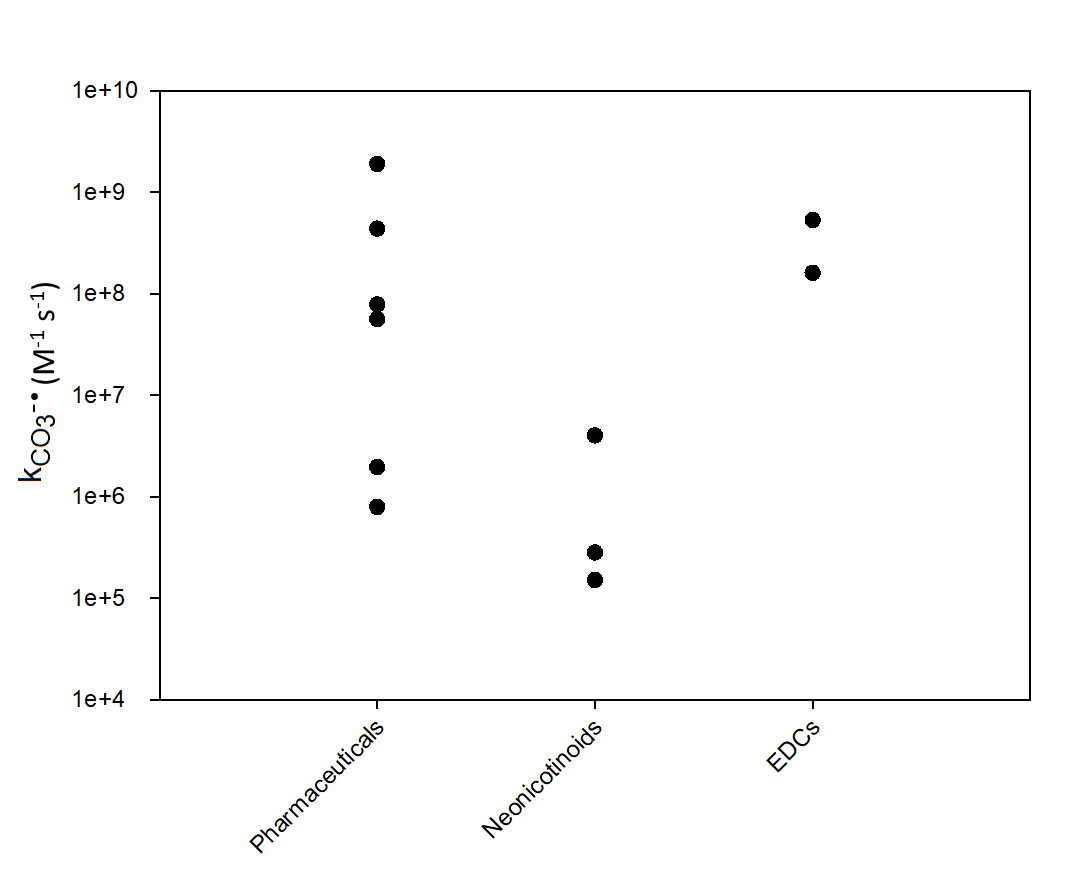


**Figure S3.** Second-order rate constants for compounds included in this work and CO_3_^-•^. The bimolecular rate constants for the compounds are categorized into the same categories as presented in the main body of the text. Pharmaceuticals: [18,29–31]; neonicotinoids: [32]; endocrine disruption chemicals (EDCs): [33].


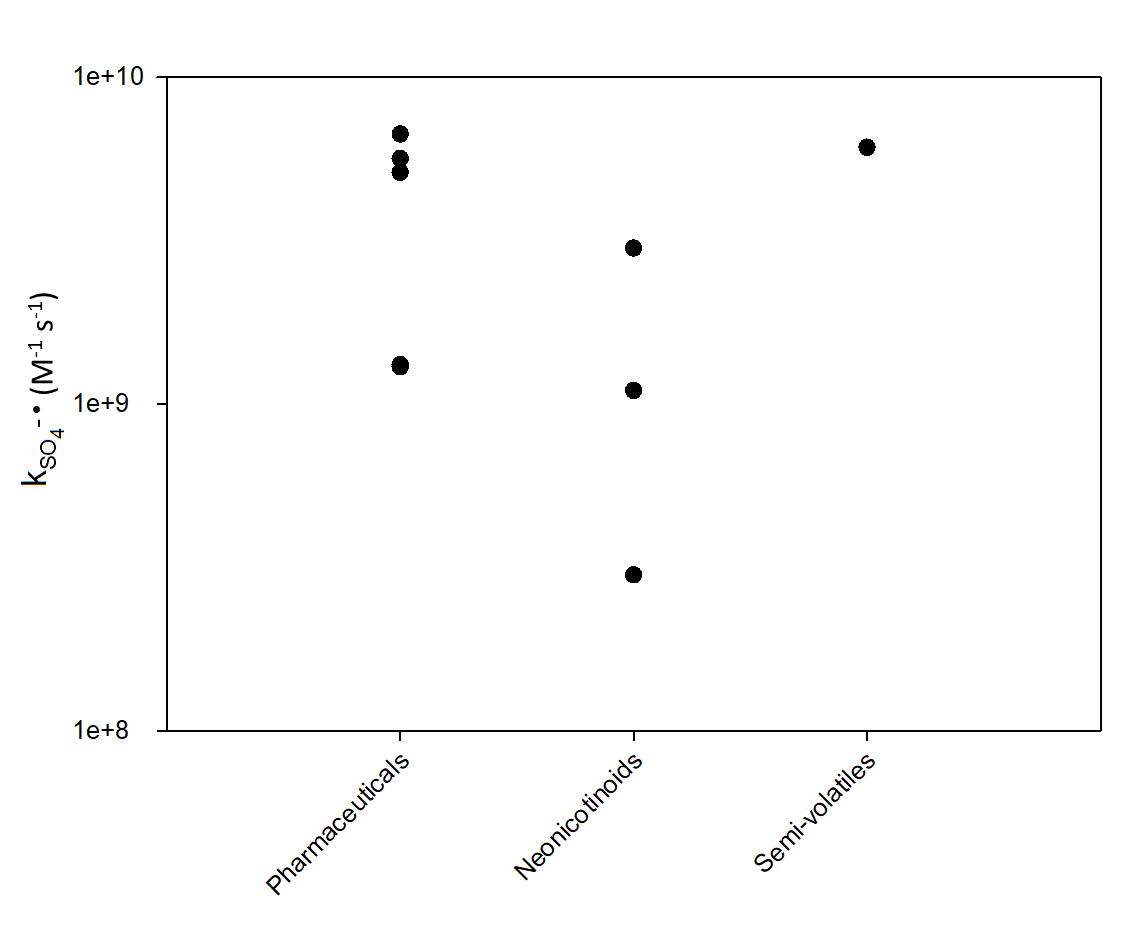


**Figure S4.** Second-order rate constants for compounds included in this work and SO_4_^-•^. The bimolecular rate constants for the compounds are categorized into the same categories as presented in the main body of the text. pharmaceuticals: [18,34]; neonicotinoids: [35] ; semi-volatile compound: [36].


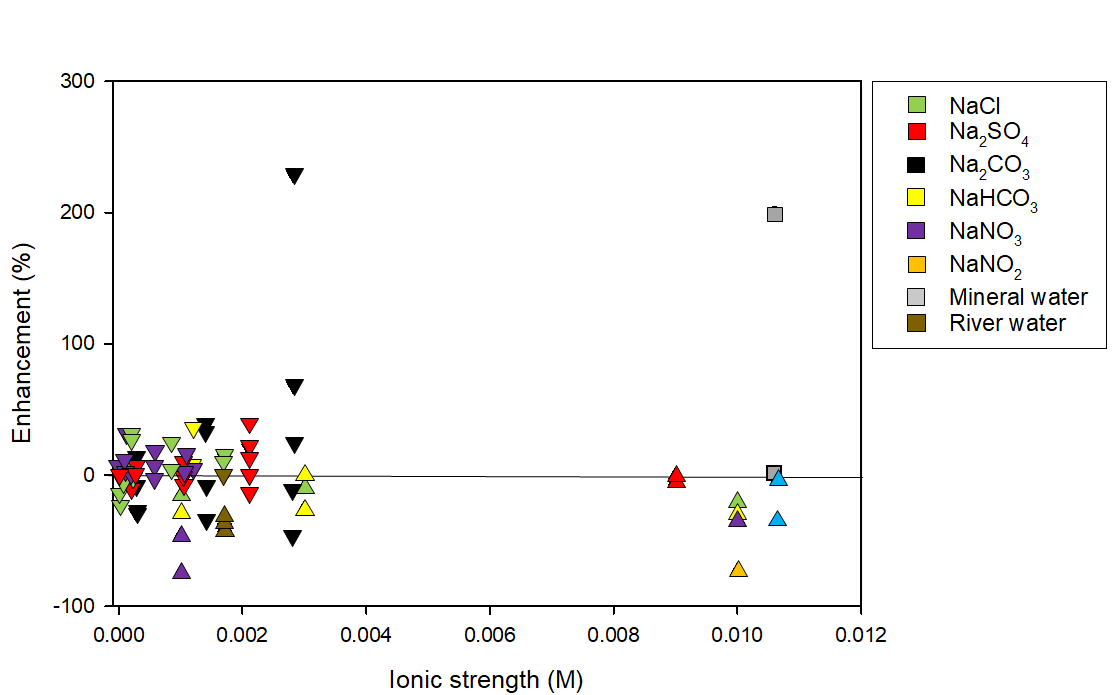


**Figure S5.** An inset, up to 0.012 M ionic strength, of the enhancement of inorganic salt type and frequency on the degradation of various dyes. The marker shape indicates ranges of frequency: ▼– 200-500 kHz; ▲ – 600-1000 kHz; ■ – 1700 kHz. The color of the symbol indicates salt type as indicated by the legend. Included references [37–42]. Refer to Table 2 for a synopsis of each study.


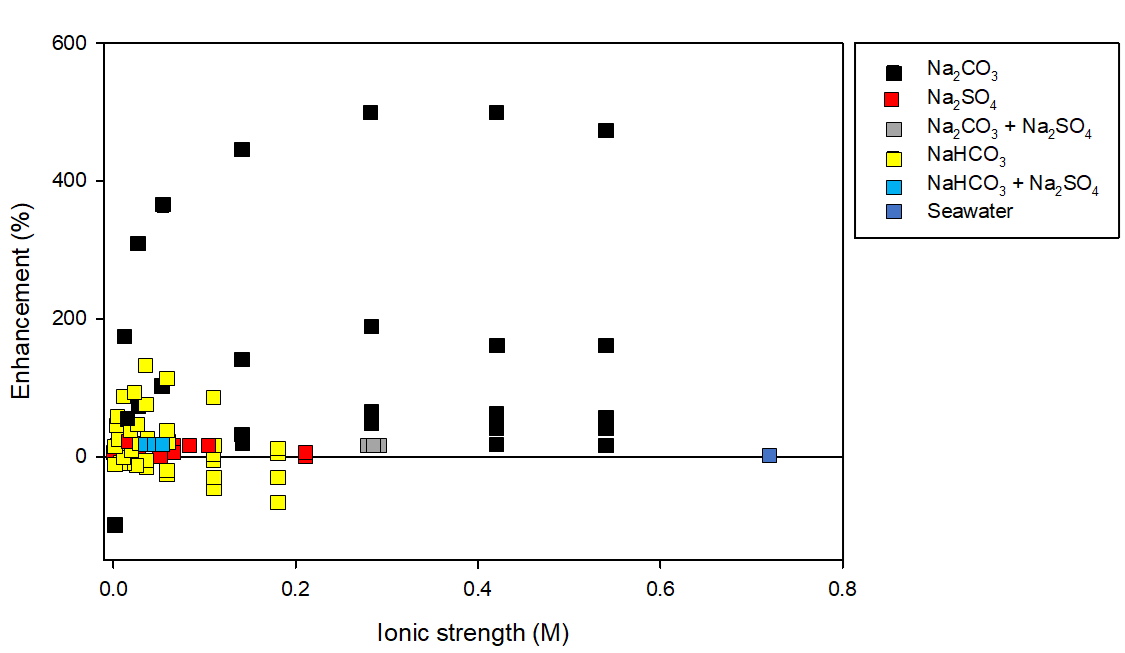


**Figure S6.** The enhancement of inorganic salt type on the dye Rhodamine B. The color of the symbol indicates salt type as indicated by the legend. Frequency: 300 kHz. Included references: [40,43,44]. Refer to Table 2 for a synopsis of each study.

**
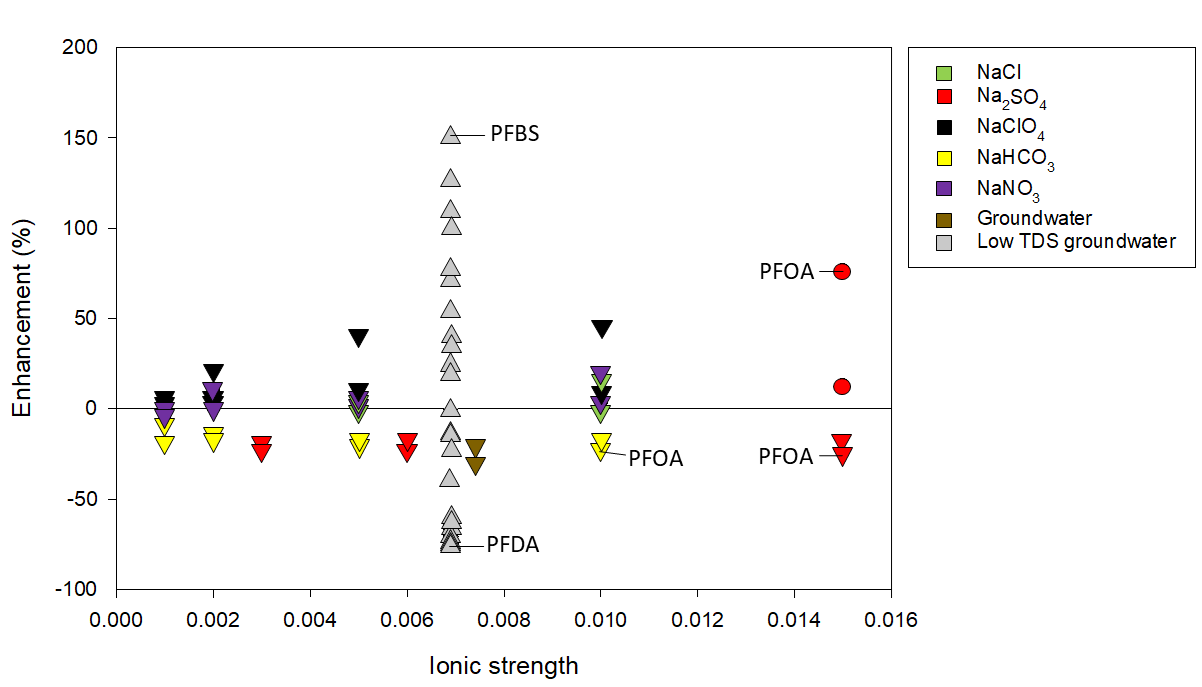
**

**Figure S7.** An inset, up to 0.016 M ionic strength, of the enhancement of inorganic salt type and frequency on the degradation of PFAS. Shape indicates specific frequency: ● – 40 kHz; ▼ – 612 kHz; ▲– 700 kHz. The color of the shape indicates salt type as indicated in the legend. Studies included: [45–47]. Refer to Table 2 for a synopsis of each study.


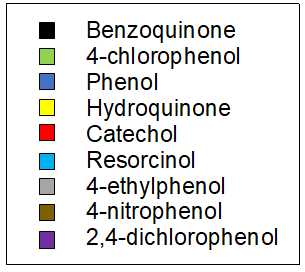

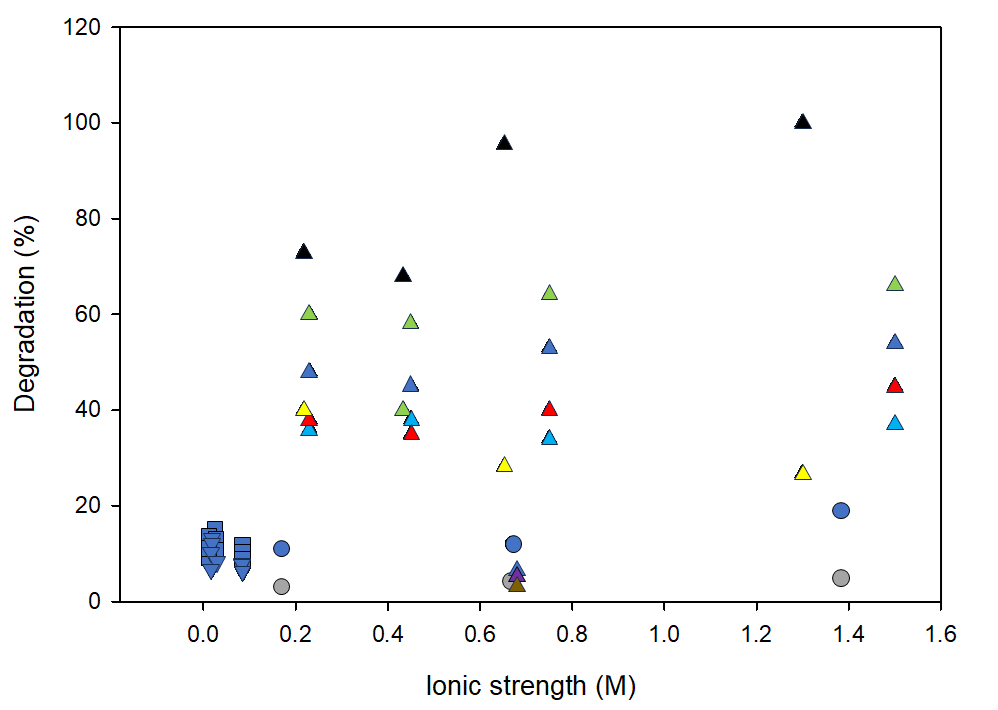


**Figure S8.** The degradation (%) of semi-volatile compounds including phenol and similar compounds. The rate constants are excluded. Phthalate ester data is excluded. Shape indicates specific frequency: ● – 20 kHz; ■ – 25 kHz; ▼ – 36 kHz; ▲ – 200 kHz. The color of the shape indicates salt type as indicated in the legend. Studies included: [48–52]. Refer to Table 2 for a synopsis of each study.

References

[1] S. Hammami, N. Bellakhal, N. Oturan, M.A. Oturan, M. Dachraoui, Degradation of Acid Orange 7 by electrochemically generated •OH radicals in acidic aqueous medium using a boron-doped diamond or platinum anode: A mechanistic study, Chemosphere 73 (2008) 678–684. https://doi.org/10.1016/j.chemosphere.2008.07.010.

[2] G. V. Buxton, C.L. Greenstock, W.P. Helman, A.B. Ross, Critical Review of rate constants for reactions of hydrated electrons, hydrogen atoms and hydroxyl radicals (⋅OH/⋅O− in Aqueous Solution, J. Phys. Chem. Ref. Data 17 (1988) 513–886. https://doi.org/10.1063/1.555805.

[3] A.A. Özcan, A. Özcan, Investigation of applicability of Electro-Fenton method for the mineralization of naphthol blue black in water, Chemosphere 202 (2018) 618–625. https://doi.org/10.1016/j.chemosphere.2018.03.125.

[4] P.R. Kulkarni, S.D. Richardson, B.N. Nzeribe, D.T. Adamson, S.S. Kalra, S. Mahendra, J. Blotevogel, A. Hanson, G. Dooley, S. Maraviov, J. Popovic, Field Demonstration of a Sonolysis Reactor for Treatment of PFAS-Contaminated Groundwater, J. Environ. Eng. 148 (2022) 1–12. https://doi.org/10.1061/(asce)ee.1943-7870.0002064.

[5] M.M. Huber, S. Canonica, G.-Y. Park, U. von Gunten, Oxidation of Pharmaceuticals during Ozonation and Advanced Oxidation Processes, Environ. Sci. Technol. 37 (2003) 1016–1024. https://doi.org/10.1021/es025896h.

[6] E.J. Rosenfeldt, K.G. Linden, Degradation of Endocrine Disrupting Chemicals Bisphenol A, Ethinyl Estradiol, and Estradiol during UV Photolysis and Advanced Oxidation Processes, Environ. Sci. Technol. 38 (2004) 5476–5483. https://doi.org/10.1021/es035413p.

[7] Y. Lei, S. Cheng, N. Luo, X. Yang, T. An, Rate Constants and Mechanisms of the Reactions of Cl• and Cl2• with Trace Organic Contaminants, Environ. Sci. Technol. 53 (2019) 11170–11182. https://doi.org/10.1021/acs.est.9b02462.

[8] H. Fang, Y. Gao, G. Li, J. An, P.-K. Wong, H. Fu, S. Yao, X. Nie, T. An, Advanced Oxidation Kinetics and Mechanism of Preservative Propylparaben Degradation in Aqueous Suspension of TiO _2_ and Risk Assessment of Its Degradation Products, Environ. Sci. Technol. 47 (2013) 2704–2712. https://doi.org/10.1021/es304898r.

[9] M.L. Dell’Arciprete, L. Santos-Juanes, A.A. Sanz, R. Vicente, A.M. Amat, J.P. Furlong, D.O. Mártire, M.C. Gonzalez, Reactivity of hydroxyl radicals with neonicotinoid insecticides: mechanism and changes in toxicity, Photochem. Photobiol. Sci. 8 (2009) 1016–1023. https://doi.org/10.1039/b900960d.

[10] H. Wang, J. Zhan, L. Gao, G. Yu, S. Komarneni, Y. Wang, Kinetics and mechanism of thiamethoxam abatement by ozonation and ozone-based advanced oxidation processes, J. Hazard. Mater. 390 (2020) 122180. https://doi.org/10.1016/j.jhazmat.2020.122180.

[11] J.K. Thomas, Rates of reaction of the hydroxyl radical, Trans. Faraday Soc. 61 (1965) 702. https://doi.org/10.1039/tf9656100702.

[12] K. Sehested, N. Getoff, F. Schwoerer, V.M. Markovic, S.O. Nielsen, Pulse radiolysis of oxalic acid and oxalates, J. Phys. Chem. 75 (1971) 749–755. https://doi.org/10.1021/j100676a004.

[13] H. Shemer, K.G. Linden, Aqueous photodegradation and toxicity of the polycyclic aromatic hydrocarbons fluorene, dibenzofuran, and dibenzothiophene, Water Res. 41 (2007) 853–861. https://doi.org/10.1016/j.watres.2006.11.022.

[14] M.E. Lindsey, M.A. Tarr, Inhibition of Hydroxyl Radical Reaction with Aromatics by Dissolved Natural Organic Matter, Environ. Sci. Technol. 34 (2000) 444–449. https://doi.org/10.1021/es990457c.

[15] A. Brotchie, T. Statham, M. Zhou, L. Dharmarathne, F. Grieser, M. Ashokkumar, Acoustic bubble sizes, coalescence, and sonochemical activity in aqueous electrolyte solutions saturated with different gases, Langmuir 26 (2010) 12690–12695. https://doi.org/10.1021/la1017104.

[16] W.R. Haag, C.C.D. Yao, Rate constants for reaction of hydroxyl radicals with several drinking water contaminants, Environ. Sci. Technol. 26 (1992) 1005–1013. https://doi.org/10.1021/es00029a021.

[17] A.L. Camargo-Perea, E.A. Serna-Galvis, J. Lee, R.A. Torres-Palma, Understanding the effects of mineral water matrix on degradation of several pharmaceuticals by ultrasound: Influence of chemical structure and concentration of the pollutants, Ultrason. Sonochem. 73 (2021). https://doi.org/10.1016/j.ultsonch.2021.105500.

[18] L. Lian, B. Yao, S. Hou, J. Fang, S. Yan, W. Song, Kinetic Study of Hydroxyl and Sulfate Radical-Mediated Oxidation of Pharmaceuticals in Wastewater Effluents, Environ. Sci. Technol. 51 (2017) 2954–2962. https://doi.org/10.1021/acs.est.6b05536.

[19] A.K. De, B. Chaudhuri, S. Bhattacharjee, B.K. Dutta, Estimation of ⋅OH radical reaction rate constants for phenol and chlorinated phenols using UV/H2O2 photo-oxidation, J. Hazard. Mater. 64 (1999) 91–104. https://doi.org/10.1016/S0304-3894(98)00225-8.

[20] G.E. Adams, B.D. Michael, Pulse radiolysis of benzoquinone and hydroquinone. Semiquinone formation by water elimination from trihydroxy-cyclohexadienyl radicals, Trans. Faraday Soc. 63 (1967) 1171. https://doi.org/10.1039/tf9676301171.

[21] W.Z. Tang, C.P. Huang, Effect of chlorine content of chlorinated phenols on their oxidation kinetics by Fenton’s reagent, Chemosphere 33 (1996) 1621–1635. https://doi.org/10.1016/0045-6535(96)00278-0.

[22] L. Ashton, G. V. Buxton, C.R. Stuart, Temperature dependence of the rate of reaction of OH with some aromatic compounds in aqueous solution. Evidence for the formation of a π-complex intermediate?, J. Chem. Soc., Faraday Trans. 91 (1995) 1631–1633. https://doi.org/10.1039/FT9959101631.

[23] J. Kochany, J.R. Bolton, Mechanism of photodegradation of aqueous organic pollutants. 2. Measurement of the primary rate constants for reaction of hydroxyl radicals with benzene and some halobenzenes using an EPR spin-trapping method following the photolysis of hydrogen peroxide, Environ. Sci. Technol. 26 (1992) 262–265. https://doi.org/10.1021/es00026a004.

[24] B.C. Gilbert, J.K. Stell, W.J. Peet, K.J. Radford, Generation and reactions of the chlorine atom in aqueous solution, J. Chem. Soc. Faraday Trans. 1 Phys. Chem. Condens. Phases 84 (1988) 3319. https://doi.org/10.1039/f19888403319.

[25] L. Wojnárovits, E. Takács, Rate constants of dichloride radical anion reactions with molecules of environmental interest in aqueous solution: a review, Environ. Sci. Pollut. Res. 28 (2021) 41552–41575. https://doi.org/10.1007/s11356-021-14453-w.

[26] X. Zhang, J. Zhai, Y. Lei, H. Huang, P. Ren, D. Lambropoulou, X. Yang, Enhanced formation of trichloronitromethane precursors during UV/monochloramine treatment, J. Hazard. Mater. 422 (2022) 126813. https://doi.org/10.1016/j.jhazmat.2021.126813.

[27] M.L. Alegre, M. Geronés, J.A. Rosso, S.G. Bertolotti, A.M. Braun, D.O. Mártire, M.C. Gonzalez, Kinetic Study of the Reactions of Chlorine Atoms and Cl2•- Radical Anions in Aqueous Solutions. 1. Reaction with Benzene, J. Phys. Chem. A 104 (2000) 3117–3125. https://doi.org/10.1021/jp9929768.

[28] D.O. Mártire, J.A. Rosso, S. Bertolotti, G.C. Le Roux, A.M. Braun, M.C. Gonzalez, Kinetic Study of the Reactions of Chlorine Atoms and Cl _2_ ^•-^ Radical Anions in Aqueous Solutions. II. Toluene, Benzoic Acid, and Chlorobenzene, J. Phys. Chem. A 105 (2001) 5385–5392. https://doi.org/10.1021/jp004630z.

[29] R.H. Bisby, N. Tabassum, Properties of the radicals formed by one-electron oxidation of acetaminophen—A pulse radiolysis study, Biochem. Pharmacol. 37 (1988) 2731–2738. https://doi.org/10.1016/0006-2952(88)90035-4.

[30] B.A. Wols, C.H.M. Hofman-Caris, Review of photochemical reaction constants of organic micropollutants required for UV advanced oxidation processes in water, Water Res. 46 (2012) 2815–2827. https://doi.org/10.1016/j.watres.2012.03.036.

[31] R. Zhang, Y. Yang, C.-H. Huang, L. Zhao, P. Sun, Kinetics and modeling of sulfonamide antibiotic degradation in wastewater and human urine by UV/H 2 O 2 and UV/PDS, Water Res. 103 (2016) 283–292. https://doi.org/10.1016/j.watres.2016.07.037.

[32] M.L. Dell’Arciprete, J.M. Soler, L. Santos-Juanes, A. Arques, D.O. Mártire, J.P. Furlong, M.C. Gonzalez, Reactivity of neonicotinoid insecticides with carbonate radicals, Water Res. 46 (2012) 3479–3489. https://doi.org/10.1016/j.watres.2012.03.051.

[33] Y. Huang, M. Kong, D. Westerman, E.G. Xu, S. Coffin, K.H. Cochran, Y. Liu, S.D. Richardson, D. Schlenk, D.D. Dionysiou, Effects of HCO3– on Degradation of Toxic Contaminants of Emerging Concern by UV/NO3, Environ. Sci. Technol. 52 (2018) 12697–12707. https://doi.org/10.1021/acs.est.8b04383.

[34] L. Wojnárovits, E. Takács, Rate constants of sulfate radical anion reactions with organic molecules: A review, Chemosphere 220 (2019) 1014–1032. https://doi.org/10.1016/j.chemosphere.2018.12.156.

[35] M.L. Dell’Arciprete, C.J. Cobos, D.O. Mártire, J.P. Furlong, M.C. Gonzalez, Reaction kinetics and mechanisms of neonicotinoid pesticides with sulfate radicals, New J. Chem. 35 (2011) 672–680. https://doi.org/10.1039/C0NJ00726A.

[36] Q. Mei, J. Sun, D. Han, B. Wei, Z. An, X. Wang, J. Xie, J. Zhan, M. He, Sulfate and hydroxyl radicals-initiated degradation reaction on phenolic contaminants in the aqueous phase: Mechanisms, kinetics and toxicity assessment, Chem. Eng. J. 373 (2019) 668–676. https://doi.org/10.1016/j.cej.2019.05.095.

[37] M.P. Rayaroth, U.K. Aravind, C.T. Aravindakumar, Effect of inorganic ions on the ultrasound initiated degradation and product formation of triphenylmethane dyes, Ultrason. Sonochem. 48 (2018) 482–491. https://doi.org/10.1016/j.ultsonch.2018.07.009.

[38] F. Guzman-Duque, C. Pétrier, C. Pulgarin, G. Peñuela, R.A. Torres-Palma, Effects of sonochemical parameters and inorganic ions during the sonochemical degradation of crystal violet in water, Ultrason. Sonochem. 18 (2011) 440–446. https://doi.org/10.1016/j.ultsonch.2010.07.019.

[39] H. Ferkous, S. Merouani, O. Hamdaoui, Sonolytic degradation of naphthol blue black at 1700 kHz: Effects of salts, complex matrices and persulfate, J. Water Process Eng. 9 (2016) 67–77. https://doi.org/10.1016/j.jwpe.2015.11.003.

[40] O. Hamdaoui, S. Merouani, Impact of seawater salinity on the sonochemical removal of emerging organic pollutants, Environ. Technol. 41 (2020) 2305–2313. https://doi.org/10.1080/09593330.2018.1564071.

[41] M.P. Rayaroth, U.K. Aravind, C.T. Aravindakumar, Ultrasound based AOP for emerging pollutants: from degradation to mechanism, Environ. Sci. Pollut. Res. 24 (2017) 6261–6269. https://doi.org/10.1007/s11356-016-6606-4.

[42] O. Hamdaoui, S. Merouani, M. Ait Idir, H.C. Benmahmoud, A. Dehane, A. Alghyamah, Ultrasound/chlorine sono-hybrid-advanced oxidation process: Impact of dissolved organic matter and mineral constituents, Ultrason. Sonochem. 83 (2022) 105918. https://doi.org/10.1016/j.ultsonch.2022.105918.

[43] S. Merouani, O. Hamdaoui, F. Saoudi, M. Chiha, Sonochemical degradation of Rhodamine B in aqueous phase: Effects of additives, Chem. Eng. J. 158 (2010) 550–557. https://doi.org/10.1016/j.cej.2010.01.048.

[44] S. Merouani, O. Hamdaoui, F. Saoudi, M. Chiha, C. Pétrier, Influence of bicarbonate and carbonate ions on sonochemical degradation of Rhodamine B in aqueous phase, J. Hazard. Mater. 175 (2010) 593–599. https://doi.org/10.1016/j.jhazmat.2009.10.046.

[45] J. Cheng, C.D. Vecitis, H. Park, B.T. Mader, M.R. Hoffmann, Sonochemical degradation of perfluorooctane sulfonate (PFOS) and perfluorooctanoate (PFOA) in groundwater: Kinetic effects of matrix inorganics, Environ. Sci. Technol. 44 (2010) 445–450. https://doi.org/10.1021/es902651g.

[46] S. Singh Kalra, B. Cranmer, G. Dooley, A.J. Hanson, S. Maraviov, S.K. Mohanty, J. Blotevogel, S. Mahendra, Sonolytic destruction of Per- and polyfluoroalkyl substances in groundwater, aqueous Film-Forming Foams, and investigation derived waste, Chem. Eng. J. 425 (2021) 131778. https://doi.org/10.1016/j.cej.2021.131778.

[47] J.C. Lin, S.L. Lo, C.Y. Hu, Y.C. Lee, J. Kuo, Enhanced sonochemical degradation of perfluorooctanoic acid by sulfate ions, Ultrason. Sonochem. 22 (2015) 542–547. https://doi.org/10.1016/j.ultsonch.2014.06.006.

[48] M.H. Uddin, B. Nanzai, K. Okitsu, Effects of Na2SO4 or NaCl on sonochemical degradation of phenolic compounds in an aqueous solution under Ar: Positive and negative effects induced by the presence of salts, Ultrason. Sonochem. 28 (2016) 144–149. https://doi.org/10.1016/j.ultsonch.2015.06.028.

[49] T. Sivasankar, V.S. Moholkar, Physical insights into the sonochemical degradation of recalcitrant organic pollutants with cavitation bubble dynamics, Ultrason. Sonochem. 16 (2009) 769–781. https://doi.org/10.1016/j.ultsonch.2009.02.009.

[50] I.M. Khokhawala, P.R. Gogate, Intensification of sonochemical degradation of phenol using additives at pilot scale operation, Water Sci. Technol. 63 (2011) 2547–2552. https://doi.org/10.2166/wst.2011.532.

[51] N.N. Mahamuni, A.B. Pandit, Effect of additives on ultrasonic degradation of phenol, Ultrason. Sonochem. 13 (2005) 165–174. https://doi.org/10.1016/j.ultsonch.2005.01.004.

[52] M.H. Uddin, K. Okitsu, Effects of Sodium Sulfate and Sodium Chloride for Sonochemical Degradation on 1,4-benzoquinone and Hydroquinone in Aqueous Solution, J. Kejuruter. 30 (2018) 161–169. https://doi.org/10.17576/jkukm-2018-30(2)-05.
